# Ligand‐Screened Cerium‐Based MOF Microcapsules Promote Nerve Regeneration via Mitochondrial Energy Supply

**DOI:** 10.1002/advs.202306780

**Published:** 2023-11-30

**Authors:** Xinzhao Jiang, Wei Wang, Jincheng Tang, Meng Han, Yichang Xu, Lichen Zhang, Jie Wu, Yiyang Huang, Zhouye Ding, Huiwen Sun, Kun Xi, Yong Gu, Liang Chen

**Affiliations:** ^1^ Department of Orthopedics The First Affiliated Hospital of Soochow University Orthopedic Institute Soochow University 188 Shizi Road Suzhou Jiangsu 215000 China; ^2^ Department of Spinal Surgery Xuzhou Central Hospital Xuzhou 221000 China

**Keywords:** cerium, metal‐organic framework, mitochondria, spinal cord injury

## Abstract

Although mitochondria are crucial for recovery after spinal cord injury (SCI), therapeutic strategies to modulate mitochondrial metabolic energy to coordinate the immune response and nerve regeneration are lacking. Here, a ligand‐screened cerium‐based metal‐organic framework (MOF) with better ROS scavenging and drug‐loading abilities is encapsulated with polydopamine after loading creatine to obtain microcapsules (Cr/Ce@PDA nanoparticles), which reverse the energy deficits in both macrophages and neuronal cells by combining ROS scavenging and energy supplementation. It reprogrames inflammatory macrophages to the proregenerative phenotype via the succinate/HIF‐1α/IL‐1β signaling axis. It also promotes the regeneration and differentiation of neural cells by activating the mTOR pathway and paracrine function of macrophages. In vivo experiments further confirm the effect of the microcapsules in regulating early ROS‐inflammation positive‐feedback chain reactions and continuously promoting nerve regeneration. This study provides a new strategy for correcting mitochondrial energy deficiency in the immune response and nerve regeneration following SCI.

## Graphical abstract

This article applies a ligand‐screened cerium‐based metal‐organic framework (MOF) with better reactive oxygen species (ROS) scavenging and drug‐loading abilities are encapsulated with polydopamine after loading creatine to obtain microcapsules (Cr/Ce@polydopamine (PDA) nanoparticles), which reverse the energy deficits in both macrophages and neuronal cells by combining ROS scavenging and energy supplementation.

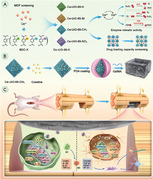

## 1 Introduction

Spinal cord injury (SCI) is a devastating neurological disease that can cause severe motor and sensory dysfunction and is a substantial financial burden on patients.^[^ ^1^ ^]^ The secondary injury phase after mechanical damage includes oxidative stress, inflammation, apoptotic signaling, and other pathological features, producing severe biological and chemical damage to spinal tissues. It is a primary target for the current treatment of SCI owing to its modifiable nature.^[^ ^2^ ^]^ As the main source of energy production, mitochondria in injured spinal cord cells undergo a series of pathophysiological changes in morphology and function in the second phase, such as mitochondrial atrophy, reduced number of cristae, excessive generation of free radicals, and decreased ATP production, which finally results in an energy‐metabolism disorder.^[^ ^3^, ^4^ ^]^ The high dependence of the immune response and neuronal‐regenerative processes on the mitochondrial metabolic state and energy makes targeting mitochondrial energy recovery a promising therapeutic strategy.^[^ ^5^ ^]^ The sequential inflammatory cascade recruits macrophages to the injury site three days after SCI. As the main effectors, a rational switch for macrophages from an inflammatory (M1) to a reparative (M2) phenotype is key to promoting the repair of injured tissue by regulating transitions through different phases of the healing response.

A sustained inflammatory response after injury affects nerve regeneration, and the M2‐secreted extracellular factors like platelet‐derived growth factor (PDGF) and neurotrophin‐3(NT3) are potent inducers of neuronal production.^[^ ^6^, ^7^ ^]^ This dynamic shift in phenotype depends on the state of cellular mitochondrial metabolism. The pro‐inflammatory environment induces a shift from oxidative to glycolytic metabolism in macrophages via activating the NF‐κB pathway, which leads to an M1 phenotype characterized by the upregulation of glycolytic and pentose phosphate pathways (PPP), disruption of the Krebs cycle, and downregulation of oxidative phosphorylation (OXPHOS).^[^ ^5^, ^8^ ^]^ Converting macrophages to the M2 phenotype by protecting mitochondrial function and reprogramming their metabolism may be a potential therapeutic strategy for SCI.^[^ ^9^ ^]^ Meanwhile, axonal growth or regeneration is an energy‐requiring process, and the regenerative capacity is positively correlated with mitochondrial activity and density at the injury site, whereas energy deficiency caused by mitochondrial dysfunction after SCI may greatly limit axonal regeneration.^[^ ^10^ ^]^ It follows that modulating the immune environment and neuroregenerative capacity in SCI through regulating mitochondrial function may aid the design of new therapeutic approaches for spinal cord regeneration.

Mitochondrial disorders after ischemia and hypoxia can stimulate the production of reactive oxygen species (ROS), further damaging mitochondrial homeostasis. Blocking the Krebs cycle of macrophages triggers lipid peroxidation, ferroptosis, and the consumption of nitric oxide, which causes secondary damage to spinal nerves.^[^ ^11^, ^12^, ^13^ ^]^ Therefore, removing ROS from the spinal cord environment and cellular mitochondria (mtROS) is important for protecting mitochondria. Compared with the low efficiency of traditional antioxidant molecules (vitamins, flavonoids, and polyphenols) and natural enzymes (catalase [CAT], superoxide dismutase [SOD], and glutathione peroxidase [GPx]), which are unstable under environmental conditions, antioxidant nanoenzymes offer a new option for ROS‐related biotherapeutics with broad‐spectrum ROS removal activity, high stability in physiological environments, and superior biocompatibility.^[^ ^14^ ^]^

Cerium nanoparticles exhibit efficient biocatalytic antioxidant properties by reversible binding of oxygen atoms and cycling between the Ce (III) and Ce (IV) states. Their feasibility has been verified in various neurological disease models, including Alzheimer's disease, cerebral hemorrhage, and stroke, which make cerium NPs a suitable candidate for clearing ROS in SCI.^[^ ^15^, ^16^, ^17^, ^18^ ^]^ However, owing to the diverse pathological mechanisms in the secondary phase of SCI, rescuing mitochondrial damage by eliminating ROS alone may be insufficient to meet the energy requirements during spinal cord recovery. Creatine is a storage form of energy that allows for the rapid regeneration of ATP from ADP in organisms. Reversing energy deficiency in SCI through direct energy supplementation with creatine can enhance axonal regeneration in mice, and maintaining normal levels of ATP during injury also helps restore mitochondrial function.^[^ ^19^, ^20^ ^]^ Combination therapy of ROS scavenging and energy supplementation may synergistically affect the recovery of mitochondrial energy metabolism in the SCI setting.

However, the hydrophilicity of creatine makes it difficult to penetrate lipid biofilms such as the blood‐brain barrier; therefore, systemic intraperitoneal or intravenous injection leads to insufficient effective concentrations at the site of injury.^[^ ^21^ ^]^ Therefore, it is necessary to introduce biological delivery vectors that synergistically regulate ROS and supply mitochondrial energy to promote nerve repair. The diverse activities of biological materials provide diverse choices of delivery carriers. Metal‐organic frameworks (MOFs), which consist of organic linkers and metal‐containing nodes, are characterized by molecular‐level catalytic centers, high porosity, and high loading capacity. The multifunctional structure of MOF gives them changeable morphologies and chemical properties, and the weak ligand bonds ensure that they are biodegradable. These features make MOFs some of the most promising materials for nanoenzyme and drug delivery.^[^ ^22^, ^23^ ^]^ The use of GelMA hydrogels to load nanoparticles allows their ordered release and their ability to mimic the natural extracellular matrix allows cells to proliferate in the GelMA scaffolds, making them suitable for spinal cord implantation.^[^ ^24^ ^]^

**Scheme 1:**
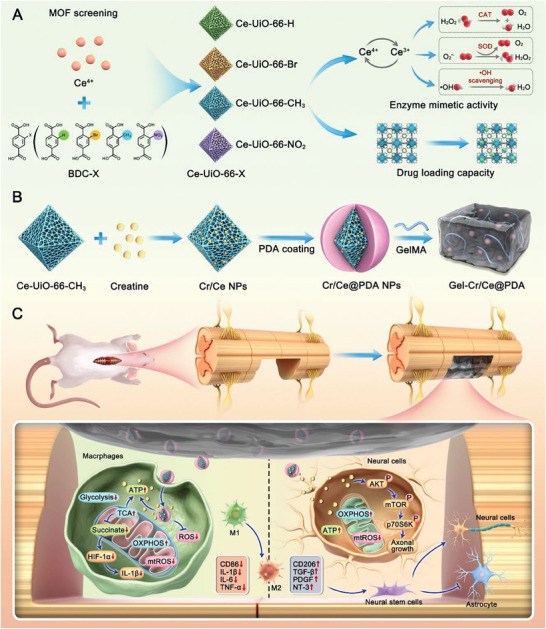
Preparation and the potential mechanisms of Gel‐Cr/Ce@PDA in treating spinal cord injury. A) Preparation and comparison of Ce‐Uio‐66‐X. B) Preparation of Gel‐Cr/Ce@PDA. C) Schematic diagram showing the mechanism Gel‐Cr/Ce@PDA promoting macrophages M2 polarization and neuronal cells regeneration.

In this study, we constructed a nanomaterial that can actively and rapidly neutralize excess ROS and supply mitochondrial energy to regulate the immune microenvironment while continuously promoting nerve regeneration. Firstly, to ensure the high catalytic effect and larger loading capacity of “external MOFs,” we used a ligand‐engineering strategy by replacing the H in the 1,4‐benzenedicarboxylic acid (BDC) ligand to modulate the biocatalytic antioxidant properties and surface area of Ce‐UiO‐66, and we have explored for the first time the antioxidant properties as well as the drug‐loading capacity of cerium MOFs. The “internal drug” creatine was encapsulated into Ce‐UiO‐66‐CH_3,_ chosen for its balance between catalytic performance and drug‐loading capacity. Subsequently, the nanoparticles were coated with polydopamine (PDA) to improve sustained drug‐release efficiency. Our nanoparticles were endocytosed like “energy pills” by macrophages during the acute inflammatory phase, and the macrophage polarization and mitochondrial metabolic status analyses revealed the switch of macrophages into anti‐inflammatory phenotypes. Joint analysis of the transcriptome and metabolomics further explored the potential mechanism by which NPs affect functional changes in macrophages. Macrophage paracrine functions affecting neural stem cell differentiation and drug release from nanoparticles to promote neurite growth have also been explored separately, demonstrating the role of NPs in nerve regeneration at later stages after SCI. Finally, these multifunctional nanoparticles were incorporated into gelatin methacrylate hydrogels and photo‐crosslinked for implantation into rat spinal cord hemisectional models to evaluate the in vivo immunomodulation and neuroregenerative properties of these novel microcapsules (**Scheme** **1**).

## 2 Results and Discussion

### 2.1 Synthesis and Characterization of Ce‐UiO‐66‐X

**Figure 1:**
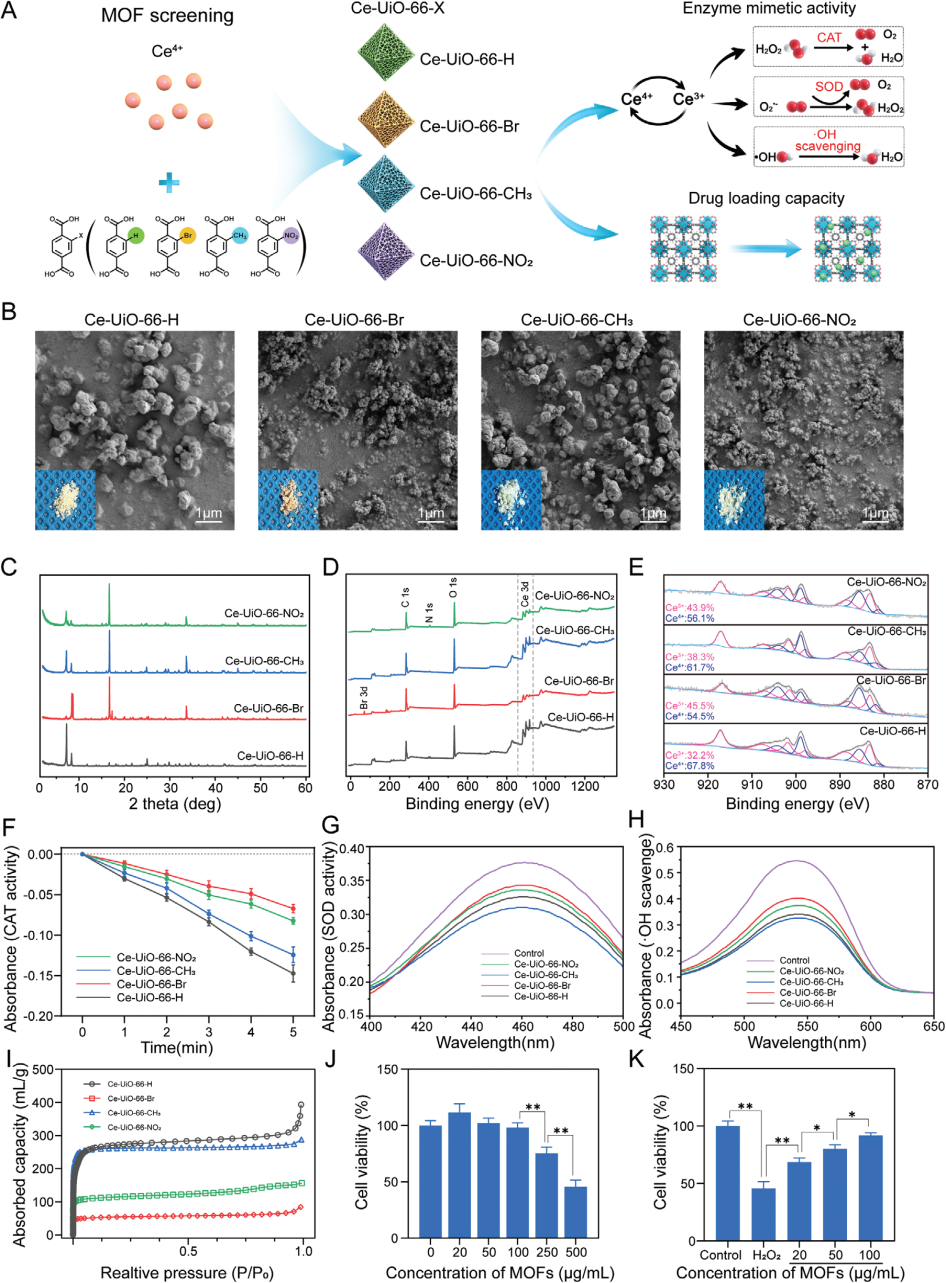
Synthesis and characterization of Ce‐UiO‐66‐X. A) Schematic diagram of preparation and comparison of Ce‐Uio‐66‐X. B) Gross morphology and SEM images of Ce‐UiO‐66‐X. C) XRD of Ce‐UiO‐66‐X. D) XPS of Ce‐UiO‐66‐X. E) High‐resolution XPS spectra of Ce in Ce‐UiO‐66‐X. F) CAT‐mimicking and G) SOD‐mimicking and H) ·OH scavenging ability of Ce‐UiO‐66‐X. I) Absorb isotherm linear plot of Ce‐UiO‐66‐X. J) Cell viability under different concentrations of Ce‐UiO‐66‐CH_3_. K) Cell protection ability of different concentrations Ce‐UiO‐66‐CH_3_ under H_2_O_2_ treatment. (*n* = 3, error bars, means ± SD; all analyses were done using one‐way ANOVA with Tukey's post hoc test *P < 0.05 and **P < 0.01).

Based on a previous study, we synthesized Ce‐UiO‐66‐X (X = H, Br, CH_3_, and NO_2_) with cerium metal nodes and different terephthalate linkers (**Figure** **1**A).^[^ ^25^ ^]^ Scanning electron microscopy (SEM) and transmission electron microscopy (TEM) images revealed the morphology of the Ce‐UiO‐66‐X MOFs; the synthesized MOF particles were of different sizes (Figure 1B and Figure S1, Supporting Information). The powder X‐ray diffraction (PXRD) patterns and Fourier transform infrared (FTIR) spectra of the Ce‐UiO‐66‐X MOFs were consistent with previous literature, demonstrating their successful preparation (Figure 1C and Figure S2, Supporting Information).^[^ ^25^ ^]^ The existence of Ce, C, and O in all MOFs was confirmed by X‐ray photoelectron spectroscopy (XPS), and Br and N were found in the Br‐ and NO_2_‐substituted MOFs (Figure 1D). The quick switch between the Ce^3+^ and Ce^4+^ states during redox reactions endows cerium NPs with high ROS‐scavenging ability, and the catalytic activity of cerium NPs is affected by the surface Ce^4^/Ce^3+ +^ ratio.^[^ ^14^ ^]^

The Ce^3+^ site is responsible for O_2_^−^ removal through SOD mimics and ·OH via redox reactions, while the Ce^4+^ site is known to decompose H_2_O_2_ through CAT mimics.^[^ ^26^, ^27^ ^]^ The Ce^4+^/Ce^3+^ ratio in particles is size‐dependent, and the fraction of Ce^4+^ increases with particle size.^[^ ^28^ ^]^ The XPS analysis of Ce in the MOFs also supported the finding that H‐ and CH_3_‐substituted MOFs with larger sizes showed higher Ce^4+^ concentrations than Br‐ and NO_2_‐substituted MOFs (Figure 1E). We then compared the catalytic activities and efficiencies of the MOFs in ROS scavenging. CAT mimetic activity was detected by monitoring the absorbance of H_2_O_2_ reacting with Ti(SO_4_)_2_ at 405 nm; the reduction at 405 nm indicated the decomposition of H_2_O_2_. Ce‐UiO‐66‐H had the highest CAT mimetic activity, attributable to its higher surface Ce^4+^ concentration (Figure 1F). Meanwhile, the SOD‐mimetic and ·OH‐scavenging abilities were measured using test kits. The xanthine (X)/xanthine oxidase (XO) system generating O_2_^−^ can reduce nitrogen blue tetrazolium to blue formazan, which can be detected at 560 nm. SOD activity was calculated based on O_2_^−^ scavenging. The content of ·OH generated via the Fenton reaction was determined with Griess reagent after administering the electron acceptor. Notably, although the proportion of Ce^3+^ on the Ce‐UiO‐66‐H and Ce‐UiO‐66‐CH_3_ surface is lower than that on the other two MOFs, they still exhibited greater SOD‐mimetic activity and ·OH scavenging. This may be attributed to the larger surface areas of Ce‐UiO‐66‐H and Ce‐UiO‐66‐CH_3_ that provide more reactive sites for O_2_^−^ and ·OH scavenging. The BET isotherms showed that CH_3_‐ (770.12 m^2^ g^−1^), Br‐ (164.29 m^2^ g^−1^), and NO_2_‐ (346.89 m^2^ g^−1^) substituted OFs had lower surface areas for the existence of functionalized linker molecules compared with Ce‐UiO‐66‐H (1055.52 m^2^ g^−1^) (Figure 1I and Table S1, Supporting Information). ─CH3 substituted MOF exhibited the highest SOD‐mimetic and ·OH‐scavenging ability among the four MOFs, possibly because its surface area was closer to that of Ce‐UiO‐66‐H and it had a higher Ce^3+^ ratio. This seems to be more in line with our needs for in vivo applications because O_2_^−^ and ·OH are directly associated with inflammatory responses.^[^ ^29^, ^30^ ^]^

The large surface area also resulted in a better drug‐loading capacity, and Ce‐UiO‐66‐H (14.40%±0.55%) and Ce‐UiO‐66‐CH_3_ (13.92%±0.32%) had larger creatine‐loading capacities than the other two MOFs (6.38% ± 0.38% of Ce‐UiO‐66‐Br and 9.24% ± 0.35% of Ce‐UiO‐66‐NO_2_). However, no significant difference was found between the CH_3_‐ and H‐substituted MOFs, possibly due to the similar micropore volume of both (0.39 mL g^−1^ of Ce‐UiO‐66‐H versus 0.38 mL g^−1^ of Ce‐UiO‐66‐CH_3_) (Table S1, Supporting Information). Therefore, the Ce‐UiO‐66‐CH_3_ with the best SOD‐mimetic and ·OH‐scavenging abilities and large drug‐loading capacity among the four MOFs was selected for further biological study. Cytotoxicity experiments were performed to assess the biocompatibility of Ce‐UiO‐66‐CH_3_ (hereinafter referred to as MOF or Ce‐MOF). After 24 h of incubation with 0–100 µg mL^−1^ MOFs, the cell viability was maintained at 90%, demonstrating the good biocompatibility of NPs at these concentrations (Figure 1J). The cytoprotective ability of the Ce‐MOF nanoenzymes against H_2_O_2_‐induced oxidative stress was investigated. After 1000‐µM‐H_2_O_2_ treatment and incubation with MOFs at different concentrations for 12 h, the cell death rate decreased with increasing concentrations of nanoenzymes, with 100 µg mL^−1^ having the highest cell viability (Figure 1K).

### 2.2 Hydrogel Scaffold Preparation and Characterization

**Figure 2:**
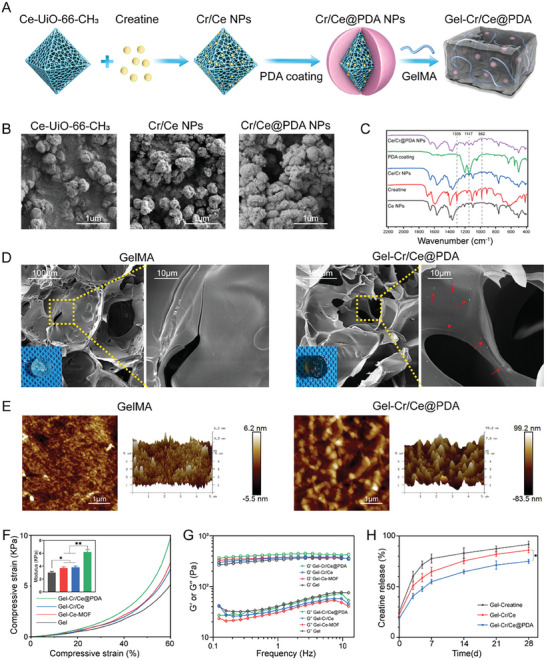
Hydrogel scaffold preparation and characterization. A) Schematic diagram of preparation of Gel‐Cr/Ce@PDA. B) SEM images of Ce‐MOF, Cr/Ce NPs, and Cr/Ce@PDA NPs. C) FTIR of Ce‐MOF, Creatine, Cr/Ce NPs, PDA, and Cr/Ce@PDA NPs. D) Gross morphology and SEM images of GelMA and Gel‐Cr/Ce@PDA. E) AFM of GelMA and Gel‐Cr/Ce@PDA. F) Stress–strain curves, compressive modulus and G) rheological analyses of the hydrogels. H) Creatine release curve of the hydrogels after incubation in saline. (*n* = 3, error bars, means ± SD; all analyses were done using one‐way ANOVA with Tukey's post hoc test *P < 0.05 and **P < 0.01).

The structure of the nanoenzyme composite is shown in **Figure** **2**A, including the Ce‐UiO‐66‐CH_3_ core, MOF‐loaded creatine, and polydopamine surface coating. SEM and TEM images revealed morphological changes before and after creatine loading and PDA modification (Figure 2B and Figure S3, Supporting Information). The average particle size increased from 264.8 nm for Ce‐MOF to 397.2 nm for Cr/Ce NPs, demonstrating the successful loading of creatine into Ce‐MOFs. The encapsulation of PDA further increased the particle size to 452.3 nm at Cr/Ce@PDA NPs. FTIR spectroscopy further demonstrated the encapsulation of creatine in the Ce‐MOF and PDA coatings (Figure 2C). In the FTIR spectra of the Cr/Ce NPs, the nanomaterials showed characteristic peaks attributable to the Ce‐MOFs, and the successful modification of creatine was evidenced by the absorption peaks associated with creatine at 1305 cm^−1^ for the carboxylic acid C‐OH telescopic vibration and 982 cm^−1^ for the C─H bending vibration. In the FTIR spectra of the Cr/Ce@PDA NPs, the composite nanomaterials showed all the characteristic peaks attributable to the Cr/Ce NPs, whereas the PDA shell modification introduced other functional groups. The large number of hydroxyl groups in the PDA molecules further increased the peak intensity of the ─OH telescoping vibration region of the composite nanomaterials, whereas the ether C─O─C bond at 1147 cm^−1^ stretching vibrations was introduced by successfully modifying the PDA shell layer. Cytotoxicity experiments were performed to assess the NP biocompatibility. After 24 h of incubation with 0–100 µg mL^−1^ Cr/Ce@PDA NPs, the cell viability was maintained at 90%, demonstrating the good biocompatibility of Cr/Ce@PDA NPs at concentrations below 100 µg mL^−1^ (Figure S4, Supporting Information).

GelMA hydrogels can mimic some essential properties of native extracellular matrix and allow cells to spread and proliferate into GelMA scaffolds.^[^ ^24^ ^]^ Nanoparticle‐loaded GelMA in the presence of a photoinitiator (lithium phenyl[2,4,6‐trimethylbenzoyl] hypophosphonate, LAP) using UV‐light exposure produced covalently crosslinked hydrogel scaffolds for implantation at the SCI lesion site. The stretching vibration peaks of ─C═C─ at 1628 cm^−1^ and 3290 cm^−1^ were reduced after photocrosslinking, indicating successful crosslinking of the hydrogels (Figure S5, Supporting Information). SEM images revealed porous microstructures with similar pore sizes of 150–300 µm for all hydrogel scaffolds, which proved its ability to work as a space for cell growth and proliferation (Figure 2D). Local magnification of the pores on the hydrogel surface showed uniformly dense nanoparticles (marked with red arrows), demonstrating effective loading. The AFM analysis revealed that the average surface roughness (Ra) of the hydrogel increased from 1.17 to 22.5 nm due to the Cr/Ce@PDA NPs (Figure 2E). Adding nanoenzymes strengthened the gelatin‐based hydrogels, resulting in a higher Young's modulus. The Gel‐Cr/Ce@PDA have the highest Young's modulus during the compression test, which could be attributed to the fact that the catechol groups of PDA in the Cr/Ce@PDA structure can be linked to GelMA through hydrogen bonding. The increase in hydrogen bonding can improve the physical cross‐linking of the GelMA, which increased Young's modulus of Gel‐Cr/Ce@PDA. These hydrogels exhibited mechanical properties similar to those of the spinal cord (<10 KPa) (Figure 2F). The frequency‐sweep test showed that the *G*′ and *G*″ of the hydrogels were almost unchanged when the testing frequency was between 0.1 and 10 Hz, suggesting their integrity. *G*′ was greater than *G*″, indicating a gelatinized state (Figure 2G). The *G*′ of Gel‐Cr/Ce@PDA was the highest at 440 Pa among the hydrogels, suggesting that adding Cr/Ce@PDA strengthened the mechanical strength of hydrogel (Figure 2G). The swelling test (Figure S6, Supporting Information) showed that all hydrogels swelled rapidly within 8 h of immersion in PBS solution and reached swelling equilibrium after 12 h of immersion. The addition of nanoparticles did not significantly affect the swelling ratio of hydrogels. Considering that the swelling ability of the hydrogels might put pressure on the spinal cords and induce additional damage to tissue regeneration, to avoid this damage, the hydrogels were soaked in PBS overnight before the surgery, and by the time it is implanted in vivo the hydrogels has been pre‐swollen.

Creatine‐release experiments demonstrated that loading creatine via MOFs showed slow‐release characteristics at an early stage (<7 d) compared to adding creatine directly to the hydrogel. However, there was no significant difference in this physisorption after three weeks. The PDA coating on the Cr/Ce NPs enabled the sustained release of creatine over a 4 week period. This promotes long‐term nerve repair (Figure 2H). The release of cerium was detected with ICP‐MS, and compared with Gel‐Ce‐MOF and Gel‐Cr/Ce, the NPs in the Gel‐Cr/Ce@PDA showed slightly slower release, which may be due to the formation of excess hydrogen bonds between PDA and GelMA (Figure S7, Supporting Information). No significant difference was observed between these hydrogels and the control group in live/dead staining, suggesting the composite materials had good biocompatibility (Figure S8, Supporting Information).

### 2.3 Nanoparticles with Mitochondrial Regulatory Functions Reprogram Macrophage Phenotype and Metabolism

The inflammatory response following SCI has been widely studied to improve prognosis. Microglia/macrophages reach their maximum number 7 d after SCI, and activating their M1 phenotype effectively removes debris from the injury; however, the release of proinflammatory compounds such as tumor necrosis factor‐α (TNF‐α), interleukin‐1β (IL‐1β), and interleukin‐6 (IL‐6) can exacerbate tissue destruction.^[^ ^31^ ^]^ Thus, the programmed conversion of the M1 phenotype to the anti‐inflammatory M2 phenotype during the acute phase of SCI contributes to SCI repair.^[^ ^32^ ^]^

**Figure 3:**
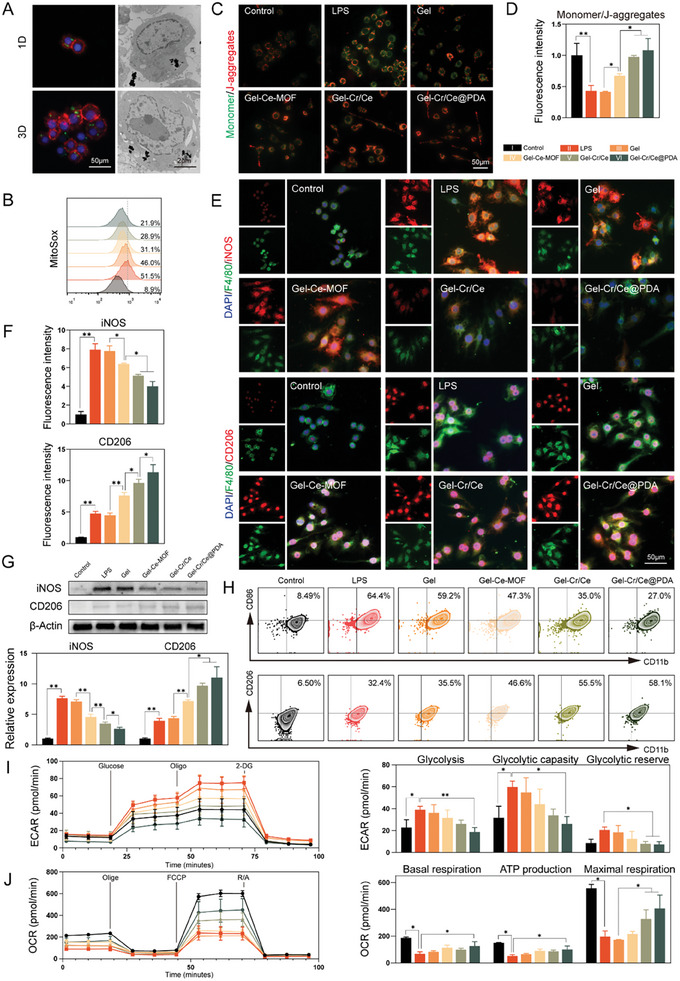
Macrophages phenotype regulation based on Gel‐Cr/Ce@PDA. A) Fluorescence and bio‐TEM images of macrophages cultured with fluorescein labeled Cr/Ce@PDA NPs for 1 and 3 d. B) Flow cytometry histograms of mitochondrial ROS levels of macrophages using MitoSOX probe. C) Detection and D) semiquantitative analysis of JC‐1 mitochondrial membrane potential of macrophages after LPS stimulation. E) Immunofluorescent images and F) semiquantitative analysis of iNOS and CD206 in macrophages cocultured with hydrogels. G) Western blot and semiquantitative analysis of iNOS and CD206 proteins expression. H) Flow cytometry histograms of CD86‐ and CD206‐positive macrophages (gated by CD11b). I) Real‐time ECARs of macrophages in the glycolysis stress test and semiquantitative analysis of glycolysis, glycolytic capacity and glycolytic reserve. J) Real‐time OCDRs of macrophages in the cell mitochondrial stress test and semiquantitative analysis of basal respiration, maximal respiration and ATP production. (*n* = 3, error bars, means ± SD; all analyses were done using one‐way ANOVA with Tukey's post hoc test *P < 0.05 and **P < 0.01).

To verify whether our material acts as an anti‐inflammatory agent, the hydrogels were cocultured with RAW264.7 macrophages. When hydrogels with fluorescein‐5‐maleimide‐labeled nanoparticles were cocultured with macrophages for 1 and 3 d, fluorescence microscopy revealed that the nanoparticles were internalized into the macrophage cytoplasm via endocytosis (**Figure** **3**A). This endocytosis was time‐dependent owing to the continuous release of nanoparticles from the hydrogel, as evidenced by the biotransmission electron microscopy images (Figure 3A). Given that regulating the macrophage‐polarization state can be achieved by regulating the mitochondrial and metabolic states of the cell,^[^ ^5^ ^]^ we investigated the protective capacity of nanoparticles on the mitochondria in inflammatory macrophages. Numerous cells undergo cellular energy dysregulation in an inflammatory environment, as evidenced by the reduced respiratory complex activity, decreased mitochondrial membrane potential, reduced mitochondrial respiration, and inhibition of ATP production. These changes are associated with excessive ROS production.^[^ ^33^ ^]^

**Figure 4:**
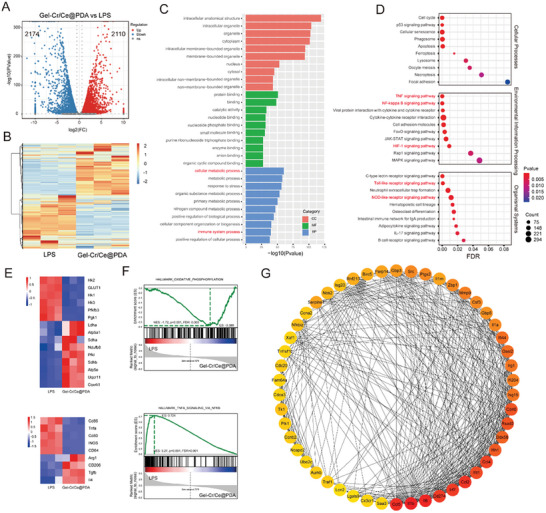
RNA sequencing analysis of macrophages with LPS induction and Gel‐Cr/Ce@PDA treatment. A) Volcano map of differentially expressed genes (DEGs) (|log2foldchange|>1, padj<0.05). B) Heatmap of DEGs. C) GO enrichment analysis of DEGs. D) KEGG enrichment analysis of DEGs in cellular processes, environmental information processing and organismal systems. E) Heatmaps of genes associated with glycolysis, OXPHOS and macrophage polarization. F) GSEA analysis of oxidative phosphorylation and TNF signaling via NF‐κB signaling pathways. G) Protein–protein interaction network Figureure (PPI) analysis of DEGs.

DCFH‐DA and MitoSox probes were used to determine the intracellular and mitochondrial ROS in macrophages. The nanoenzyme showed excellent catalytic performance in cells, with a gradual decrease in the fluorescence intensity of DCFHDA‐labeled cells (Figure S9, Supporting Information) and a gradual decrease in the proportion of Mitosox‐labeled cells (**Figure** **4**B). JC‐1 probes were used to detect mitochondrial membrane potentials in macrophages. More monomeric JC‐1 (green fluorescence) was observed in LPS‐treated macrophages, whereas a decrease in green fluorescence after coculture with Ce‐MOF indicated a decrease in mitochondrial depolarization after inflammation. Subsequent semi‐quantification showed that the synergistic effect of Ce‐MOFs and creatine significantly elevated the MMP levels in macrophages (Figure 3C,D). Furthermore, in an analysis of macrophage mitochondrial nanostructures in the LPS and Gel‐Cr/Ce@PDA groups, the proportion of swollen or ruptured mitochondria was significantly reduced in the Gel‐Cr/Ce@PDA group (Figure S10, Supporting Information). In conclusion, Gel‐Cr/Ce@PDA potentially protects macrophage mitochondria in an inflammatory environment.

To further validate the immunomodulatory ability of Gel‐Cr/Ce@PDA, the polarized phenotype of macrophages after co‐culturing with the material was assessed by immunofluorescence staining, western blotting, and flow cytometry. The proinflammatory M1 phenotype makers mainly include CD86, tumor necrosis factor‐alpha (TNF‐α), inducible nitric oxide synthase (iNOS), and interleukin (IL)−1β, whereas M2 phenotype markers include arginase‐1 (Arg‐1), CD206, and IL‐10. As shown in the immunofluorescence images of the LPS, Gel‐Ce‐MOF, Gel‐Cr/Ce, and Gel‐Cr/Ce@PDA groups, the fluorescence intensities of the M1‐specific indicator iNOS and M2‐specific indicator CD206 gradually decreased and increased, respectively (Figure 3E). iNOS and CD206 protein expression evaluated via western blotting (WB) analysis obtained similar results (Figure 3F). Flow cytometric analysis was also used to identify the polarization phenotypes of macrophages; CD11b was used to identify macrophages, CD86 was used to label the M1 subtype, and CD206 was used to label the M2 subtype. The decreased proportion of CD86 ^+^ and increased proportion of CD206 ^+^ cells suggested an increased proportion of reparative M2 macrophages after treatment with multifunctional nanoenzymes (Figure 3H and Figure S11, Supporting Information). Meanwhile, the encapsulation of both creatine and Ce‐MOF in Cr/Ce@PDA can improved the efficacy of delivery of creatine to macrophages in the early inflammatory period and reduced the loss of creatine in the environment. We cultured LPS‐induced inflammatory macrophages using Cr/Ce@PDA versus equal concentrations of Ce@PDA and creatine, and found that macrophages with Cr/Ce@PDA exhibited lower INOS and higher CD206 fluorescence intensity, which indicated that simultaneous encapsulation of creatine and Ce‐MOF in Cr/Ce@PDA can promote macrophage M2 anti‐inflammatory phenotype better than using the two alone (Figure S12, Supporting Information).

As the center of cellular metabolism, the regulation of mitochondrial function is usually accompanied by changes in the metabolic state. changes in the metabolism of macrophages are essential for switching the immune phenotype (the M1 phenotype is characterized by the upregulation of glycolytic and pentose phosphate pathways (PPP), disruption of the Krebs cycle, and downregulation of OXPHOS; the M2 phenotype is characterized by the upregulation of OXPHOS and fatty acid oxidation (FAO)).^[^ ^8^, ^34^ ^]^ To investigate whether the materials can influence the metabolic state of macrophages by regulating the functional state of mitochondria and, ultimately, the macrophage phenotype, seahorse extracellular flux analysis was used to investigate the metabolic state of inflammatory macrophages in response to Gel‐Cr/Ce@PDA. According to the glycolytic stress assay, the real‐time extracellular acidification rate (ECAR) after sequential injections of glucose, oligomycin (Oligo), and 2‐deoxy‐d‐glucose (2‐DG) exhibited macrophage glycolytic parameters (Figure 3I). Compared to the control group, glycolysis, glycolytic capacity, and glycolytic reserve were significantly increased in LPS‐triggered inflammatory macrophages. However, in the Gel‐Ce‐MOF, Gel‐Cr/Ce, and Gel‐Cr/Ce@PDA groups, the glycolytic parameters of macrophages exhibited a gradual downward trend (Figure 3I). Similarly, the real‐time oxygen‐consumption rate (OCR) in response to sequential treatment with oligo, carbonyl cyanide p‐trifluoromethoxyphenylhydrazone (FCCP), and rotenone was recorded to calculate the OXPHOS parameters of macrophages according to the cell mitochondrial‐stress test (Figure 3J). The LPS group exhibited significantly lower basal respiration, ATP production, and maximal respiration than the control group (Figure 3J), whereas the OXPHOS parameters of the macrophages gradually rebounded in the Gel‐Ce‐MOF, Gel‐Cr/Ce, and Gel‐Cr/Ce@PDA groups. Western blotting was performed to analyze the expression levels of glycolysis and OXPHOS marker proteins (Figure S13, Supporting Information). The decreased expression of glycolytic marker proteins (HK2 and GLUT1) in the LPS group was significantly elevated after treatment with Gel‐Cr/Ce@PDA (*p* < 0.05), and the protein expression levels of the complex I subunit (NDUFB8), complex II subunit (SDHA), complex IV subunit (COXIV), and complex V subunit (ATP 5A) in the Gel‐Cr/Ce@PDA group were significantly higher than those in the LPS group (*p* < 0.05). This is broadly in line with our findings, although no significant difference was observed in the complex III subunit (UQCRC1, *p* > 0.05).

Overall, the above results demonstrated that incubation with Gel‐Cr/Ce@PDA resulted in the metabolic reprogramming of inflammatory macrophages from glycolysis to oxidative phosphorylation, which may shift the macrophage phenotype. Both cerium dioxide and creatine alone can promote the shift in the macrophage phenotype from M1 to M2, but our results suggest that combining the two produces a better synergistic effect.^[^ ^15^, ^35^ ^]^ Restoring the mitochondrial membrane potential and shifts in cellular metabolic phenotypes in inflammatory macrophages suggest that this may be related to the recovery of mitochondrial function. The Impairment of mitochondrial function in inflammatory macrophages decreases their intracellular ATP concentration and shifts the metabolic phenotype toward glycolysis. In addition to cerium nanoparticle scavenging of ROS, creatine supplementation enhances cellular energetics via an energy buffer that restores cellular ATP independent of mitochondria, thus preventing the overloading of the mitochondrial respiratory chain and creating a buffer zone for mitochondrial recovery while replenishing cellular energy.^[^ ^36^, ^37^ ^]^ Thus, the two exhibit synergistic effects and encapsulating polydopamine improves the drug‐delivery efficiency and balances the M1/2 macrophage polarization.

To further investigate the regulatory mechanism of Gel‐Cr/Ce@PDA on the polarization phenotype and metabolic status of the macrophages, transcriptomic and metabolomic analyses were performed on macrophages from the LPS and Gel‐Cr/Ce@PDA groups. The principal component analysis showed significant separation between these two groups, indicating a significant difference between them (Figure S14A, Supporting Information). As shown in the differentially expressed gene (DEGs) analysis, 2110 DEGs were upregulated, and 2174 DEGs were downregulated in the Gel‐Cr/Ce@PDA group compared to the LPS group (DEGs, |log_2_foldchange| > 1, padj < 0.05)(Figure 4A,B). The DEGs were collected to perform Gene Ontology (GO) database analysis, which is typically classified into three categories: biological process (BP), molecular function (MF), and cellular component (CC), with the top ten enriched terms of each category being presented (Figure 4C).

The genes were enriched in cellular metabolic and immune system processes. This may correlate with the mechanism by which the material regulates the macrophage immune phenotype through its metabolic state. Similarly, the Kyoto Encyclopedia of Genes and Genomes (KEGG) was used to explore the underlying signaling pathways, and the top ten enriched pathways of cellular processes, environmental information processing, and organismal systems are presented (Figure 4D). Inflammatory macrophage activation‐related pathways (i.e., tumor necrosis factor (TNF), nuclear factor kappa light chain enhancer of activated B cells (NF‐kappa B), hypoxia‐inducible factor 1 (HIF‐1), nucleotide‐binding oligomerization domain (NOD)‐like receptor, and toll‐like receptor (TLR) signaling pathways) were affected, and they were all downregulated in Gel‐Cr/Ce@PDA group^[^ ^38^, ^39^ ^]^(Figure S14B, Supporting Information). The heatmap shows significantly altered expression profiles of the major genes associated with glycolysis and OXPHOS, which were downregulated or upregulated, respectively, in the Gel‐Cr/Ce@PDA group (Figure 4E). Thermographic analysis of macrophage polarization markers revealed a switch from the M1 to M2 phenotype in response to material intervention (Figure 4E). Gene‐set‐enrichment analysis (GSEA) based on hallmark gene sets was performed. TNF signaling via NF‐kappa B was down‐regulated and oxidative phosphorylation was upregulated in Gel‐Cr/Ce@PDA group, consistent with our previous research results (Figure 4F). The top DEGs were introduced into the STRING database and Cytoscape to further investigate the interactions at the protein level, and the color depth of each node was positively correlated with the degree of proteins interacting with others (Figure 4G). The abundance links were directed to the C─C motif chemokine ligand (CCL5), interleukin‐1 beta (IL‐1β), and interleukin‐6 (IL‐6), which were responsible for the pro‐inflammatory effects in M1 macrophages.^[^ ^38^ ^]^

**Figure 5:**
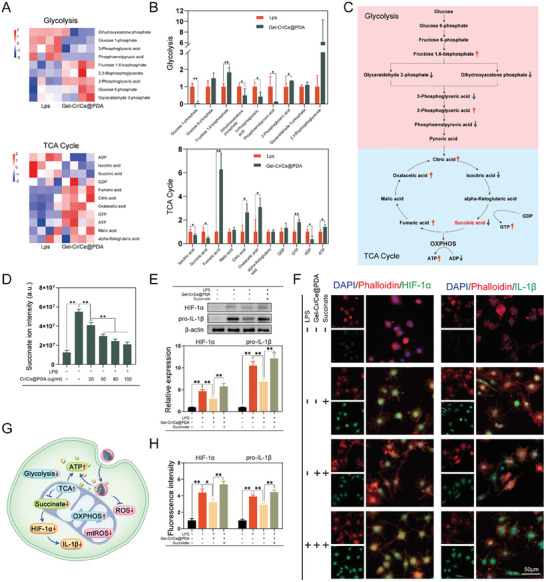
Metabolomic analysis and mechanism of Gel‐Cr/Ce@PDA promoting macrophages M2 polarization. A) Heatmaps of the glycolysis and TCA cycle related metabolites (*n* = 4). B) Quantitative analysis of glycolysis and TCA cycle related metabolites (*n* = 4). C) Schematic diagram of the changes in the target metabolites involved in the glycolysis and TCA cycle. D) Succinate levels of LPS‐induced macrophages after incubation with different concentrations of Cr/Ce@PDA NPs. E) Western blot and semiquantitative analysis of HIF‐1α and IL‐1β proteins expression. F) Immunofluorescent images of HIF‐1α and IL‐1β and semiquantitative analysis H) in macrophages. G) Schematic diagram showing the mechanism Gel‐Cr/Ce@PDA promoting macrophages M2 polarization. (*n* = 3, error bars, means ± SD; unpaired two‐tailed t test was used in (B), the other analyses were done using one‐way ANOVA with Tukey's post hoc test *P < 0.05 and **P < 0.01).

Liquid chromatography‐targeted mass spectrometry (LC‐MS/MS) was used to investigate the associated metabolites in the macrophages of the LPS and Gel‐Cr/Ce@PDA groups, and 428 intracellular metabolites were upregulated, while 179 intracellular metabolites were downregulated in the Gel‐Cr/Ce@PDA group compared with the LPS group (|log2foldchange| > 1, padj < 0.05) (Figure S15A,B, Supporting Information). The top 20 KEGG pathways enriched with differentially expressed metabolites included the TCA cycle, oxidative phosphorylation, and glycolysis, consistent with our previous cellular experiments and transcriptomic results (Figure S15C, Supporting Information). The metabolites involved in glycolysis and the TCA cycle are presented in **Figure** **5**A,B. Heatmap and quantitative analysis showed decreased levels of several key products of glycolysis, such as glyceraldehyde 3‐phosphate, 3‐phosphoglyceric acid, dihydroxyacetone phosphate, and phosphoenolpyruvic acid. Regarding the TCA cycle, the levels of citric acid, oxaloacetic acid, and fumaric acid, and the energy sources ATP and GTP increased in the Gel‐Cr/Ce@PDA group. In contrast, the level of succinic acid, which indicates a break in the TCA cycle, was significantly decreased.^[^ ^40^ ^]^ These results further demonstrate that incubation with Gel‐Cr/Ce@PDA shifts the macrophage metabolic state from pro‐inflammatory‐related glycolysis to an anti‐inflammatory‐related TCA cycle and simultaneously restores mitochondrial ATP production.

An inflammatory environment stimulates the accumulation of succinate in macrophages. Succinate dehydrogenase, a key enzyme in the tricarboxylic acid cycle and oxidative phosphorylation, can oxidize succinate to fumarate to reduce succinate accumulation.^[^ ^40^, ^41^, ^42^ ^]^ Succinate accumulation stabilizes the hypoxia‐inducible factor HIF‐1α by inhibiting the function of prolyl hydroxylase structural domain (PHD) enzyme activity. Activating HIF‐1α mediates the metabolic reprogramming of macrophages towards the M1 phenotype, promoting macrophage glycolysis and the production of inflammatory IL‐1β.^[^ ^42^, ^43^ ^]^ Combining the transcriptomic and metabolomic results, we, therefore, speculate that Gel‐Cr/Ce@PDA promotes succinate metabolism in the TCA cycle by restoring mitochondrial function and then inhibits the succinate/HIF‐1α/IL‐1β signaling axis by attenuating succinate accumulation in cells while regulating macrophage metabolism and inflammatory phenotype shift towards M2.

We measured succinate levels in LPS‐induced macrophages after incubation with different concentrations of Cr/Ce@PDA NPs via LC‐MS and found that the level of succinate decreased with increasing concentrations of nanoparticles (Figure 5D). The heatmap in transcriptomics suggested that most HIF‐1α target genes and HIF‐1α itself were downregulated in the Gel‐Cr/Ce@PDA group compared with the LPS group (Figure S16, Supporting Information). Protein expression detected by WB revealed that Gel‐Cr/Ce@PDA reduced the LPS‐induced pro‐IL‐1β and HIF‐1α, but they were significantly increased again after adding succinate (Figure 5E), evidenced via immunofluorescence and semi‐quantification (Figure 5F,H). Extracellular‐flux analysis showed that the addition of succinate reversed the metabolic regulation of the nanomaterials, inhibited macrophage OXPHOS, and promoted glycolysis (Figure S17, Supporting Information). Notably, SDH‐mediated succinate oxidation appears to be harmful because it can increase the expression of pro‐inflammatory factors through ROS production.^[^ ^44^ ^]^ However, we did not find a pro‐inflammatory effect following the reduced level of succinate via oxidation, presumably because the sustained ROS‐scavenging effect of the nanoenzymes partially counteracted oxidative‐stress‐induced inflammation, promoting the anti‐inflammatory phenotype of macrophages mainly through reducing succinate accumulation. Overall, these findings indicate that Gel‐Cr/Ce@PDA may promote the metabolic and inflammatory phenotype switch in macrophages through the Succinate/HIF‐1α/IL‐1β signaling axis.

### 2.4 Polarized Macrophages Mold Pro/Anti‐Inflammatory Microenvironment, Affecting Neural Stem Cell Differentiation

NSCs are recruited to the site of injury following SCI, where they proliferate and differentiate into neurons, astrocytes, and oligodendrocytes.^[^ ^45^ ^]^ A suitable microenvironment is essential for the proper differentiation of NSCs, and the paracrine effect of macrophages is a major source of cytokines that alter the lesion environment.^[^ ^31^ ^]^ To investigate the long‐term effects of the immune microenvironment influenced by polarized macrophages on NSC differentiation, we used a Transwell coculture system and polarized macrophages as well as NSCs. M1 polarized macrophages were transplanted into the top chamber 12 h after stimulation with LPS (the control group was not stimulated) and cultured with different hydrogels to remove the interference from polarizing factors, whereas NSCs were cultured in the bottom chamber. After 5 d of coculture, Elisa kits were used to detect the concentrations of inflammatory cytokines (TNF‐α, IL‐6) in the medium and those of growth factors TGF‐β, PDGF, and NT3, paracellularly secreted by M2 macrophages, promoting the differentiation of neural stem cells into neurons.^[^ ^7^ ^]^

**Figure 6:**
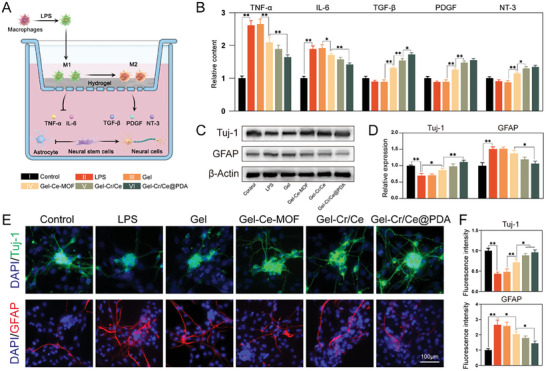
M2 polarized macrophages affect neural stem cell differentiation. A) Schematic diagram of Macrophages/NSCs transwell co‐culture system. B) Expression patterns of inflammatory and growth factors in the co‐culture system detected by ELISA. C) Western blot and D) semiquantitative analysis of Tuj‐1 and GFAP proteins expression. E) Immunofluorescent images and (F)semiquantitative analysis of Tuj‐1 and GFAP in macrophages. (*n* = 3, error bars, means ± SD; all analyses were done using one‐way ANOVA with Tukey's post hoc test *P < 0.05 and **P < 0.01).

Quantitative analysis showed that compared with the LPS and Gel groups, the concentrations of the two inflammatory cytokines were significantly reduced in the nanoparticle‐loaded hydrogel groups, with the greatest decrease in the Gel‐Cr/Ce@PDA group (**Figure** **6**B). The growth factor concentration measurements showed the opposite trend, with increasing concentrations in the nanoparticle‐loaded hydrogel groups (Figure 6B). This suggests that the regulation of macrophage polarization phenotypes by the material successfully regulates the surrounding immune microenvironment. NSCs in the bottom chamber were collected, and the levels of the neuronal differentiation marker β3‐tubulin (Tuj‐1) and astrocyte marker glial fibrillary acidic protein (GFAP) were measured by WB (Figure 6C,D) and immunofluorescence (Figure 6E,F) to assess the neurogenic differentiation. In the LPS and Gel groups, NSCs exhibited high GFAP and low Tuj‐1 fluorescence intensity, suggesting the formation of glial scars in the inflammatory microenvironment dominated by M1 macrophages. In the hydrogel group with added nanoparticles, the inhibitory inflammatory effect of M2 macrophages with paracrine growth factors increased Tuj‐1 expression and decreased GFAP expression, which indicated the promotion of neuronal differentiation. These results suggest that the Gel‐Cr/Ce @PDA‐regulated macrophage polarization affects neural stem cell differentiation through the immune microenvironment.

### 2.5 Gel‐Cr/Ce@PDA Regulates Neuronal Mitochondrial Dysfunction and Promotes Axonal Outgrowth

**Figure 7:**
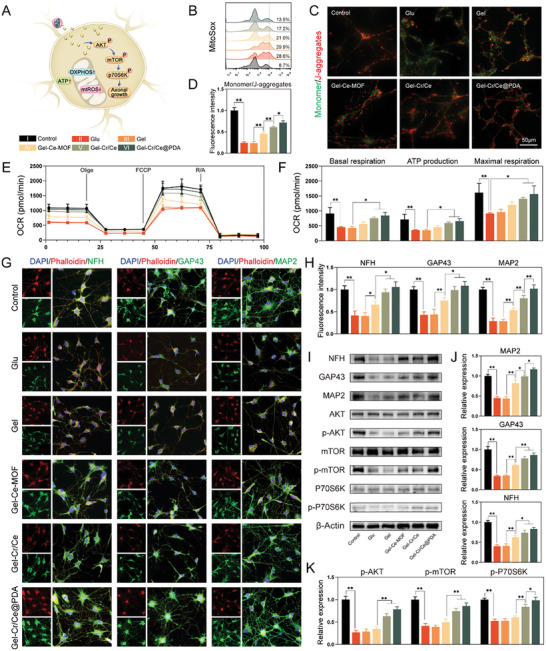
Gel‐Cr/Ce@PDA promotes axon outgrowth in the case of mitochondrial injury in neuronal cells. A) Schematic diagram Gel‐Cr/Ce@PDA promote axon growth through mTOR pathway. B) Flow cytometry histograms of mitochondrial ROS levels of neuronal cells using MitoSOX probe. C) Detection and D) semiquantitative analysis of JC‐1 mitochondrial membrane potential of neuronal cells after glutamate stimulation. E) Real‐time OARs of neuronal cells in the cell mitochondrial stress test and F) semiquantitative analysis of basal respiration, maximal respiration and ATP production. G) Immunofluorescent images and H) semiquantitative analysis of NFH, GAP43 and MAP2 in neuronal cells. I) Western blot and J,K) semiquantitative analysis of NFH, GAP43 and MAP2 protein and the relative protein of the mTOR pathway. (*n* = 3, error bars, means ± SD; all analyses were done using one‐way ANOVA with Tukey's post hoc test *P < 0.05 and **P < 0.01).

Initial SCI triggers the release of excess glutamate into the extracellular space, causing excitotoxicity, which induces excess calcium ion influx into neurons, leading to increased ROS production and mitochondrial dysfunction prior to neuronal cell death.^[^ ^5^ ^]^ Therefore, we added glutamate to the medium to simulate postinjury neuronal mitochondrial dysfunction. After seven days of co‐culture of glutamate‐treated neuronal cells with the material, DCFH‐DA and MitoSox probes were used to determine the intracellular and mitochondrial ROS in neurons. The results showed that the nanoenzymes were also effective in reducing ROS production caused by neuronal glutamate excitotoxicity, with a gradual decrease in fluorescence intensity in DCFHDA‐labeled cells (Figure S18, Supporting Information) and a gradual decrease in the proportion of Mitosox‐labeled cells (**Figure** **7**B). Similarly, the mitochondrial membrane potential assay showed that Gel‐Cr/Ce@PDA gradually restored the glutamate‐induced decrease in neuronal MMP (Figure 7C,D).

Moreover, we used Seahorse extracellular‐flux analysis to investigate mitochondrial respiration (OCR assay) in neuronal cells. The results showed that Gel‐Cr/Ce@PDA antagonized the detrimental effects of glutamate and restored basal and maximal respiration (Figure 7E). The ATP‐production assay also demonstrated the combined effects of Gel‐Cr/Ce@PDA on the recovery of the axonal energy supply, essential for the regeneration of axons (Figure 7F). Neurofilament heavy chain (NFH), growth‐associated protein‐43 (GAP43), and microtubule‐associated protein 2 (MAP2) were used to assess regenerative axon growth. The immunofluorescence staining images showed a significantly reduced fluorescence intensity of these neurogenic markers in neuronal cells with mitochondrial dysfunction. The intensity increased after the protection of mitochondrial function by scavenging ROS and increased again after combined energy supplementation, showing a synergistic effect of Gel‐Cr/Ce@PDA (Figure 7G,H). The protein levels of the three neurogenic markers assessed by WB also supported this result (Figure 7I,J). By observing axon outgrowth in each group, the Gel‐Cr/Ce@PDA group was found to have the longest axons (Figure S19, Supporting Information). We also cocultured neural cells with different concentrations of cerium MOF and creatine to explore the synergistic effect of Ce‐MOF and creatine on nerve regeneration. 100 µg mL^−1^ of Ce‐MOF was the optimal dose we derived from our cell viability assay, and 20 µg mL^−1^ of creatine exceeded the drug‐loading capacity of Ce‐UiO‐66‐CH_3_ (13.92% ± 0.32%). From the immunofluorescence and quantitative analysis, it was concluded that the expression of NFH increased with increasing doses of Ce‐MOF and creatine in the range of 100 µg mL^−1^ of Ce‐MOF and 20 µg mL^‐1^ of creatine (Figure S20, Supporting Information). In the cell uptake assay, we cocultured fluorescently labeled MOFs with neural cells for three and seven days, however, we found that there was no phagocytosis of nanoparticles by neuronal cells at three days, and that only a very small amount of nanoparticles were phagocytosed by a few neuronal cells at seven days, with the majority of neuronal cells not phagocytosing nanoparticles (Figure S21, Supporting Information). In contrast to the macrophage uptake essay, we believed that nanoparticles rely primarily on scavenging ROS and drug release in the cellular environment to aid the growth of neural cells.

Taken together, these results indicate that Gel‐Cr/Ce@PDA promotes axonal outgrowth and the formation of neural synaptic networks. Reversing energy deficits in SCI by combining the enhancement of mitochondrial transport capacity with creatine supplementation promotes axonal regeneration; our study found that removing ROS, a source of mitochondrial damage, with the application of an energy buffer also synergistically meets the needs of axonal regeneration.^[^ ^46^ ^]^ The mammalian target of the rapamycin (mTOR) pathway plays a vital role in SCI recovery because of its role in neuronal cell metabolism, growth, and proliferation.^[^ ^47^ ^]^ However, the role of mTOR signaling in nerve regeneration remains controversial. mTOR inhibition can inhibit new protein synthesis to promote axon regeneration, while other researchers have suggested that supplementation with metabolic substrates could increase OXPHOS and mTOR‐driven energy metabolism in neuronal cells, enhancing neuronal axonal branching.^[^ ^48^, ^49^ ^]^ Although the mechanism by which creatine promotes spinal cord axonal regeneration remains unknown, creatine activates the mTOR pathway in the hippocampus to increase BDNF expression during antidepressant treatment.^[^ ^50^ ^]^ Therefore, to investigate the potential mechanisms of axonal growth, we measured the relative protein expression of the AKT/mTOR signaling pathway (Figure 7I,K). The relative expression levels of p‐AKT/AKT, p‐mTOR/mTOR, and p‐P70S6K/P70S6K increased in the NP‐supplemented group, and adding creatine resulted in a larger increase. This validated our hypothesis that Gel‐Cr/Ce@PDA promotes axonal outgrowth by activating the mTOR pathway. To further verify our hypothesis, rapamycin was used to inhibit mTOR phosphorylation in the neuronal cells of the Gel‐Cr/Ce@PDA group to downregulate the mTOR pathway. Immunofluorescence and WB showed that the protein expression of NFH, GAP43, and MAP2 significantly decreased in the rapamycin group (Figure S22, Supporting Information), further supporting our hypothesis that Gel‐Cr/Ce@PDA promotes axonal regeneration via the AKT/mTOR pathway.

### 2.6 Gel‐Cr/Ce@PDA Improved Locomotor and Urinary Function Recovery in SCI Rats

**Figure 8:**
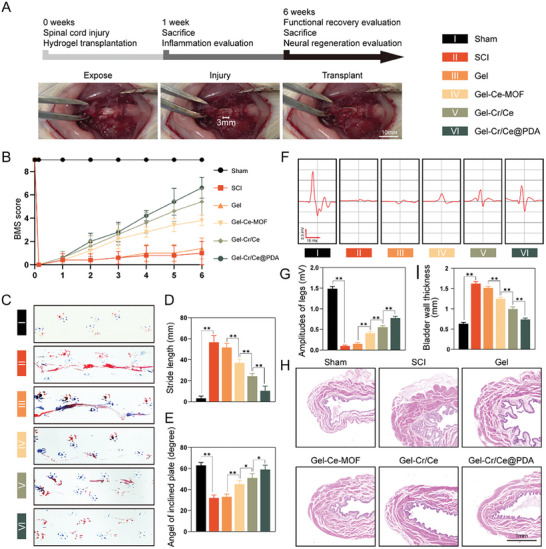
Functional recovery of rats undergoing spinal cord injury in different groups. A) Schematic diagram of the experiment timeline. B) BMS score for assessment of motor function recovery of the hindlimb. C) Representative footprints used to assess the recovery of hindlimb motor function. Forelimb footprints are shown in blue and hindlimb footprints in red, and the D) quantitative analysis of stride length in footprints. E) Angles in the inclined plane test. F) Electrophysiological analyses and G) the quantification of MEPs amplitudes from rats in different groups. H) H&E staining of bladders and (I)the measurements of the maximum bladder wall thickness. (*n* = 6, error bars, means ± SD; all analyses were done using one‐way ANOVA with Tukey's post hoc test *P < 0.05 and **P < 0.01).

A semi‐sectioned SCI model with injury at T9 in Sprague‐Dawley (SD) rats was used to explore the curative effect of Gel‐Cr/Ce@PDA in vivo. A diagram of the experimental timeline is shown in **Figure** **8**A. The Basso mouse scale (BMS) score was used to evaluate the recovery of hind‐limb motor dysfunction after SCI (Figure 8B). After hemi‐transection of the right spinal cord, the animal showed complete paralysis of the right hind limb. From two weeks postoperatively, statistically significant differences in motor recovery were observed between the treatment groups, with the hydrogel loaded with nanoparticles showing improved recovery compared with the SCI and Gel groups, indicating the early therapeutic effect of nanozymes. Four weeks postoperatively, the combined effect of the nanozymes and creatine appeared, exhibiting improved motor recovery beyond that of the Gel‐Ce‐MOF group. The recovery difference between the Gel‐Cr/Ce and group at week 6 showed the contribution of PDA encapsulation to the long‐lasting effect of Gel‐Cr/Ce@PDA.

The footprint analysis (Figure 8C,D) and inclined‐plane test (Figure 8E) results at six weeks postoperatively were consistent with the BMS scores. Rats in the Gel‐Cr/Ce@PDA group exhibited a clearer footprint with a shorter stride distance and a greater tilt angle in the inclined‐plane test, suggesting improved grip strength. Electrophysiological analyses were performed to detect motor‐evoked potentials (MEPs), which reflect functional recovery (Figure 8F,G). Six weeks postoperatively, changes in MEP signal amplitude were recorded in the gastrocnemius muscle after excitation of the spinal cord above the injury site with a stimulating electrode. The MEP signal amplitudes in the SCI and Gel groups sharply decreased compared with those in the sham group; however, with the combination treatment of Ce‐MOF and creatine, the MEP signal amplitude progressively increased, indicating the recovery of the translesional circuitry. We assessed the urinary function six weeks postoperatively through changes in bladder structure (Figure 8H,I). The results showed a reduced bladder wall thickness in the Gel‐Cr/Ce@PDA group compared to that in the SCI group, suggesting that Gel‐Cr/Ce@PDA alleviated bladder remodeling after SCI.

### 2.7 Gel‐Cr/Ce@PDA Regulates Immune Microenvironment In Vivo

**Figure 9:**
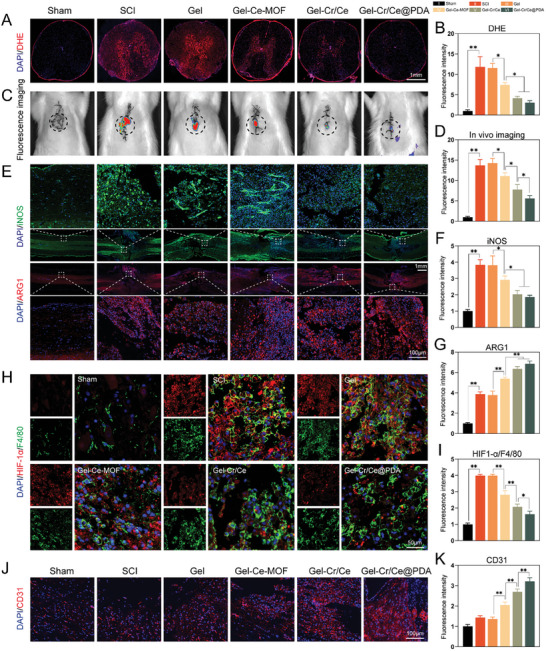
Gel‐Cr/Ce@PDA suppresses early inflammation after SCI in vivo. A) Fluorescence staining images and B) semiquantitative analysis of DHE. C) Fluorescence imaging of SCI rats after injection of ROS probe for in vivo imaging and D) semiqualitative analysis of fluorescence intensity. E) Immunofluorescent images and F,G) semiquantitative analysis of iNOS and ARG1. H) Immunofluorescent images and I) semiquantitative analysis of CD31. J) Immunofluorescent images and K) semiquantitative analysis of the ratio of HIF1‐α/F4/80 around the lesion site. (*n* = 3, error bars, means ± SD; all analyses were done using one‐way ANOVA with Tukey's post hoc test *P < 0.05 and **P < 0.01).

The suppression of inflammation at an early stage after SCI can reduce the damage caused by secondary insults to the spinal cord, thereby providing a good regenerative environment for nerve repair. Macrophages/microglia can reach maximum numbers at the lesion site 7 –10 d after SCI; thus, the rats in each treatment group were observed for short‐term acute inflammatory responses seven days after surgery.^[^ ^31^ ^]^ Dihydroethidium (DHE) (**Figure** **9**A,B) and a ROS probe (Figure 9C,D) were used for in vivo imaging to detect superoxide anion levels at the lesion site. Fluorescent semiquantitative analysis showed that neurons and macrophages produced higher levels of ROS after SCI compared to the sham group, which was the main cause of mitochondrial dysfunction in the spinal cord. The nanoenzymes catalyze the decomposition of toxic ROS, which, combined with creatine, shows synergistic effects and effectively alleviates oxidative stress.

To assess the polarization of macrophages, we performed immunofluorescence staining for iNOS (M1 phenotype marker) and ARG1 (M2 phenotype marker) in the rat spinal cords (Figure 9E–G). The combination therapy of the nanoenzymes and creatine significantly reduced the expression of iNOS and increased the expression of ARG1, and the encapsulation of nanoparticles via polydopamine in the Gel‐Cr/Ce@PDA group improved the drug‐delivery efficiency and further enhanced the beneficial effects. This suggests that Gel‐Cr/Ce@PDA facilitated the reprogramming of the macrophage phenotype and exhibited good inflammatory regulation. To investigate whether the mechanism by which the material regulates macrophage polarization in vivo is consistent with our in vitro experiments, we used immunofluorescence to label HIF1‐α and F4/80 around the injury. F4/80 was used to label the macrophages recruited to the injury site (Figure 9J,K). The decrease in the fluorescence intensity ratio of HIF1‐α/F4/80 further demonstrated that the reprogramming of macrophages was associated with the HIF signaling pathway.

Post‐traumatic angiogenesis ensures nutritional support for neuronal regeneration. Inhibition of oxidative stress and modulation of the immune microenvironment promotes the endogenous repair of the microvasculature and regeneration of axons after SCI.^[^ ^51^, ^52^ ^]^ Therefore, we quantified the expression of CD31 (platelet endothelial cell adhesion molecule) around the lesion site (Figure 9H,I); the enhanced expression of CD31 indicated that Gel‐Cr/Ce@PDA promoted blood vessel maturation via ROS clearance and inflammation regulation, providing an excellent guarantee for tissue regeneration.

### 2.8 Gel‐Cr/Ce@PDA Enhanced Neural Regeneration In Vivo

**Figure 10:**
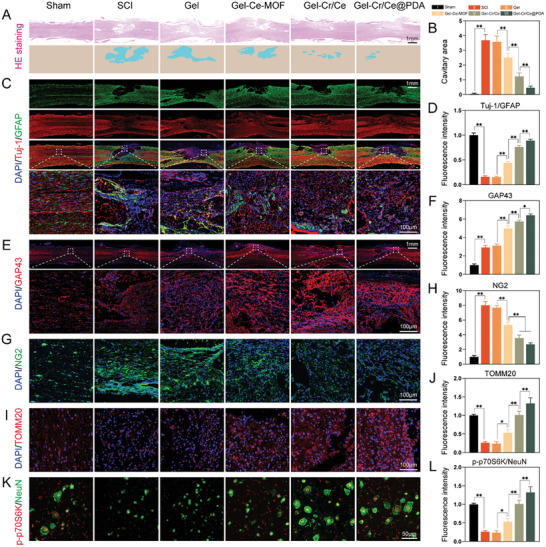
Gel‐Cr/Ce@PDA promotes neuronal regeneration after SCI in vivo. A) H&E staining of the longitudinal sections of the spinal cords and the reconstructed spinal cord longitudinal section, and red‐colored areas represent the cavitary areas. B) Quantitative analysis of the cavitary areas. C) Immunofluorescent images and D) semiquantitative analysis of the ratio of Tuj‐1/GFAP in the lesion site. E) Immunofluorescent images and F) semiquantitative analysis of GAP43. G) Immunofluorescent images and H) semiquantitative analysis of NG2. I) Immunofluorescent images and J) semiquantitative analysis of TOMM20. K) Immunofluorescent images and L) semiquantitative analysis of the ratio ofp‐p70S6K/NeuN in the lesion site. (*n* = 3, error bars, means ± SD; all analyses were done using one‐way ANOVA with Tukey's post hoc test *P < 0.05 and **P < 0.01).

The spinal cords of the rats were collected six weeks after SCI to investigate the pathological changes after Gel‐Cr/Ce@PDA treatment further. In the HE‐stained transverse spinal cord sections, the cavitary area was 3.68 ± 0.32 mm^2^ in the SCI group; however, there was a gradual decrease in the cavitary area of the spinal cords; the lesion volume was the smallest in the Gel‐Cr/Ce@PDA group (0.46 ± 0.07 mm^2^) (**Figure** **10**A,B). This demonstrated that the hydrogel scaffolds provided a good platform for cell growth and promoted tissue formation. The distribution of neurons and astrocytes at the lesion site was analyzed via double staining for Tuj‐1 and GFAP (Figure 10C,D). In the SCI and Gel groups, astrocytes accumulated mainly at the lesion edge to form scars; however, in the groups treated with added nanoparticles, a decrease in astrocytes and an increase in neural cells were observed. Semi‐quantitative analysis showed that the Tuj‐1/GFAP fluorescence‐intensity ratio was highest in the Gel‐Cr/Ce@PDA group, demonstrating that Gel‐Cr/Ce@PDA effectively enhanced neuronal regeneration and inhibited astrocyte proliferation.

Immunofluorescent staining of the neuromodulin GAP43 protein, which regulates neuronal growth, was also used to evaluate the outcomes of neural regeneration (Figure 10E,F), and the chondroitin sulfate proteoglycan NG2 protein was used to mark the glial scar tissue in nerve regeneration (Figure 10G,H). Significantly higher numbers of GAP43‐positive cells were observed at the lesion site in the Gel‐Cr/Ce@PDA group than in the other groups, and the fluorescence intensity of NG2 was significantly reduced, further illustrating the ability of Gel‐Cr/Ce@PDA to reduce inflammation‐induced scar formation after SCI, removing the obstruction to nerve and blood vessel growth and promoting nerve regeneration. The immunofluorescence staining of the mitochondrial outer membrane marker TOMM20 at the lesion site was significantly higher in the Gel‐Cr/Ce@PDA group than in the other groups, indicating an increased mitochondrial mass, which might be in line with the neuroprotective and neuronal modulation effects of Gel‐Cr/Ce@PDA (Figure 10I,J). Consistent with our in vitro experiments, the upregulation of specific hallmarks of the mTOR pathway p‐p70S6K suggested that Gel‐Cr/Ce@PDA activated the mTOR anabolic pathway, which is associated with neuronal regeneration (Figure 10K,L). In addition, the histological analysis confirmed no significant changes in pathological abnormalities in major organs (i.e., heart, liver, lung, and kidney) in rats treated with hydrogels compared with the control group (Figure S23, Supporting Information), which indicating that these hydrogels had excellent biocompatibility as nerve repair materials.

In addition to free radical damage. Mitochondrial dysfunction after SCI is also involved in neuronal cell and oligodendrocyte death, such as necrosis, apoptosis, and ferroptosis. Regulation of mitochondrial dynamics and biogenesis, as well as partial mitochondrial uncoupling and mitochondrial transplantation to increase the number of mitochondria, etc., are also promising targets for spinal cord injury regeneration, which are desirable for future work.

## 3 Conclusion

In summary, targeting the recovery of mitochondrial energy metabolism via a combination of ROS scavenging and energy supplementation had a synergistic effect in the treatment of SCI. The ligand‐screened cerium‐based MOFs exhibited better ROS‐scavenging and drug‐loading abilities and were loaded with creatine to synthesize Gel‐Cr/Ce@PDA. Gel‐Cr/Ce@PDA can regulate macrophage phenotypes towards M2 via metabolic reprogramming, which releases cytokines to form an anti‐inflammatory microenvironment promoting NSC differentiation into neuronal cells. A long‐term supply of energy also accelerates the regeneration of axons. Therefore, this study revealed a novel strategy targeting energy‐metabolism recovery in SCI and systematically explored the underlying mechanisms to provide a theoretical reference for further clinical applications.

## 4 Experimental Section

### Synthesis of Ce‐UiO‐66‐X

The synthesis of Ce‐UiO‐66‐X used a modified version of previously reported. In a 10 mL Pyrex glass reaction tube, 0.23 mmol linker (1,4‐benzendicarboxylic acid (BDC), 2‐methyl‐1,4‐benzenedicarboxylic acid (BDC‐CH_3_), 2‐bromo‐1,4‐benzenedicarboxylic acid (BDC‐Br) and 2‐nitro‐1,4‐benzenedicarboxylic acid (BDC‐NO_2_)) (Aladdin, China) and 0.5333 mmol cerium (IV) ammonium nitrate (Aladdin, China) was mixed in 1.2 mL N,N‐dimethylformamide (DMF) (Aladdin, China). Then the sealed glass reactor heated with stirring at 100 °C for 30 min. After cooling to room temperature, the final product was collected by centrifugation and washed three times with acetone.

### Creatine Loading

10 mg Ce‐UiO‐66‐X was added to 10 mL creatine solution (10 × 10^−3^ m) (Aladdin, China) and the solution was gently mixed overnight. The product was collected by centrifugation. To determine the drug loading efficiency of each Ce‐UiO‐66‐X, the residual creatine concentration in the total supernatant was measure by creatine test kit (Solarbio, China) and calculate the loading content of creatine as following equation:

(1) $$\begin{array}{ccc} \mathrm{Loading}\;\mathrm{content} & = & \frac{\mathrm{mass}\;\mathrm{of}\;\mathrm{creatine}\;\mathrm{used}-\mathrm{mass}\;\mathrm{of}\;\mathrm{residual}\;\mathrm{creatine}}{\mathrm{mass}\;\mathrm{of}\;\mathrm{Cr}/\mathrm{Ce}\;\mathrm{NPs}}\\ & & \;*100\% \end{array}$$

### PDA Coating

Tris‐HCl (10 × 10^−3^ m, pH = 8.5) (Aladdin, China) was use to dissolve dopamine hydrochloride (1 mg mL^−1^) (Aladdin, China) with gentle stirring. After 5 min, the nanoparticles (10 mg) were added into the dopamine solution the initiate the reaction. The reaction was allowed to processed for 5, 10, 20 min, then the product was collected by centrifugation and purified with water for 3 times, stored at 4 °C for future use.

### Preparation of Gel‐Cr/Ce@PDA

GelMA (60% substitution, EFL, China) and deionized water containing photoinitiator in a 10% GelMA solution were prepared. Cr/Ce@PDA NPs were directed added into the GelMA solution with a concentration of 100 µg mL^−1^ and stirred overnight. Then the hydrogel solution was photo‐crosslinked with UV irradiation (365 nm, 6.9 mW cm^−2^)

### Material Characterization

The morphology of nanoparticles mentioned above were characterized transmission electron microscopy (TEM, Hitachi, Japan) at an accelerating voltage of 100 kV and scanning electron microscopy (SEM, Hitachi, Japan) at an accelerating voltage of 10 kV. The surface roughness of the hydrogel was analyzed by atomic force microscopy (AFM, Bruker, Germany). The crystal structure of the Ce‐Uio‐66‐X was analyzed using an X‐ray diffractometry (XRD, Bruker, Germany). The X‐ray photoelectron spectroscopy (XPS, Thermo Fisher, USA) and Fourier transform infrared spectroscopy (FTIR, Thermo Fisher, USA) were also collected.

### Catalase Assay

A pathological concentration of 100 × 10^−6^ m H_2_O_2_ and Ce‐UiO‐66‐X (10 µg mL^−1^) were mixed together for 5 min. Catalase decomposition of H_2_O_2_ was rapidly arrested by the addition of ammonium molybdate, and the remaining H_2_O_2_ interacted with the ammonium molybdate to produce a yellowish complex, the variable of which was measured at 405 nm with a UV–vis spectrophotometer.

### Superoxide Dismutase (SOD) Assay

The Ce‐UiO‐66‐X (10 µg mL^−1^) nanozymes were mixed with X (0.15 × 10^−3^ m) and XO (0.02 U mL^−1^) for 5 min, then WST‐1 were added into the mixed solution. O_2_^−^ can react with WST‐1 to produce the formazan that can be detected at 450with a UV‐vis spectrophotometer.

### Hydroxyl Radical Scavenging Assay

100 × 10^−6^ m H_2_O_2_ produces hydroxyl radicals by Fenton reaction, after 5 min of co‐incubation with Ce‐UiO‐66‐X (10 µg mL^−1^), the Griess reagent was added into the mixed solution and the amount of hydroxyl radicals is proportional to the absorbance value at 550 nm.

### Swelling Ratio

To evaluate the swelling ratio of the hydrogels, the samples (*n* = 3) were freeze‐dried, weighed (W_1_) and immersed in PBS solution (pH = 7.4) at 37 °C for different time intervals (1, 2, 4, 8, 12, 24, and 48 h). And the samples were weighed (*W*_2_) and swelling ratio was measured, based on Equations (2).

(2) $$\mathrm{Swelling}\;\mathrm{ratio}=\frac{W_{2}-W_{1}}{W_{1}}*100$$

### Creatine and Cerium Release

1 g Gel‐Cr/Ce@PDA, Gel‐Cr/Ce and Gel‐Creatine which contain 20 mg creatine were immersed in 30 mL of 1% BSA in a 50 mL centrifuge tube. The tube was placed in a constant temperature vibrator at 37 °C at 100 cycles min^−1^. At time points (0.5, 3, 5, 7, 14 and 28 d), the release buffer was collected from the centrifuge tube and the concentrations of creatine in the release buffer were measured using a creatine test kit (Solarbio, China).

1 g Gel‐Cr/Ce@PDA, Gel‐Cr/Ce and Gel‐Ce‐MOF which load a total of 100 µg Ce MOF were immersed in 30 mL of 1% BSA in a 50 mL centrifuge tube. The tube was placed in a constant temperature vibrator at 37 °C at 100 cycles min^−1^. At time points (1, 3, 5, 7 and 14 d), the release buffer was collected from the centrifuge tube and resuspended in 10 mL of pure water (under nitrogen protection). The obtained supernatants were analyzed using ICP‐MS to determine the concentration of elemental cerium in each solution.

### Cell Culture

Primary neuron stem cells (NSCs) were isolated from the brain of E14 fetal rats, which was dissociated into small cell clumps with mechanical shearing under sterile conditions. The meninges and blood vessels were removed from the tissue suspension and the remaining cells were transferred to six‐well plates at 1 × 107 cells per well. Cells were cultured in complete culture medium for rat neurons (Procell, China).

RAW 264.7 line was purchased from the ATCC cell bank and cultured in high glucose DMEM medium (Gibco, USA) containing 10% FBS (Gibco, USA), which was replaced every 3 d.

### Cell Viability and Proliferation Assay

Cells were plated into 24‐well plates (1 × 10^4^ cells per well) and cocultured with each hydrogel. The viability was calculated using live/dead cell staining after three days of cocultivation. The live/dead cell working staining solution (Invitrogen, USA) was added to double‐stain cells for 15 min in the dark at 37 °C and the images of living/dead cells were captured using a fluorescence microscope (Carl Zeiss, USA). In addition, CCK‐8 reagent (Dojindo, Japan) was added to the cell culture plates at a ratio of 1:10 to detect cell proliferation on days 1, 3 and 7. 100 µL of supernatant was transferred to a 96‐well plate and measured at 450 nm using an enzyme labeling instrument after 4 hours incubation (Thermo Fisher, USA).

### Immunofluorescence Analysis

Cells or tissues were fixed in 4% paraformaldehyde for 20 min and then incubated in 0.2% Triton‐100X (Sigma‐Aldrich, USA) for 15 min. Samples were subsequently blocked with 5% BSA (Beyotime, China) overnight. The relevant primary antibody including anti‐CD206 antibody (ab64693, Abcam, USA), anti‐iNOS antibody (ab178945, Abcam, USA), anti‐F4/80 antibody (ab6640, Abcam, USA), anti‐HIF‐1α antibody (ab308433, Abcam, USA), anti‐IL‐1β antibody (ab254360, Abcam, USA), anti‐Tuj‐1 antibody (ab18207, Abcam, USA), anti‐GFAP antibody (ab7260, Abcam, USA), anti‐NFH antibody (ab207176, Abcam, USA), anti‐GAP43 antibody (ab75810, Abcam, USA), anti‐MAP2 antibody (ab5392, Abcam, USA) were then added to the samples at 4 °C overnight. Secondary antibody and DAPI were incubated with samples at 37 °C for 1 h the next day. Wash the sample with pbs three times before each operation for 5 min each time. Images were captured with a fluorescence microscope (Carl Zeiss, USA).

### Western Blot

Total cell proteins were extracted with RIPA lysis buffer (Beyotime, China) containing protease inhibitor (Beyotime, China) and phosphatase inhibitor (Beyotime, China), and the BCA protein concentration assay kit (Solarbio, China) was used for the qualification of the protein concentrations. Equal amounts of protein (10 µg) were separated with 10% SDS‐PAGE gels (Beyotime, China) and then transferred to polyvinylidene fluoride membranes. After blocked with 5% skim milk for 1 h, the membranes were incubated with primary antibody including anti‐CD206 antibody (ab64693, Abcam, USA), anti‐iNOS antibody (ab178945, Abcam, USA), anti‐HIF‐1α antibody (29547, SAB, USA), anti‐IL‐1β antibody (32165, SAB, USA), anti‐Tuj‐1 antibody (A17913, Abclonal, China), anti‐GFAP antibody (ab7260, Abcam, USA), anti‐NFH antibody (ab207176, Abcam, USA), anti‐GAP43 antibody (ab75810, Abcam, USA), anti‐MAP2 antibody (ab5392, Abcam, USA), anti‐AKT antibody (21054, SAB, USA), anti‐p‐AKT antibody (11054, SAB, USA), anti‐mTOR antibody (41187, SAB, USA), anti‐p‐mTOR antibody (C91983Bio, SAB, USA), anti‐P70S6K antibody (33240, SAB, USA), anti‐p‐P70S6K antibody (ab2571, Abcam, USA), anti‐β‐actin antibody (ab8227, Abcam, USA) at 4 °C overnight, followed by incubation with secondary antibody for 1 h the next day. The protein band were visualized using an enhanced chemiluminescence kit (Beyotime, China) and analyzed using ImageJ software.

### Flow Cytometry

Cells were cocultured with each hydrogel in a 12‐well plate at a concentration of 1 × 10^5^ per well. After 3 d of culture, cells were harvested and incubated with corresponding antibody or reagent working solutions at 27 °C for 30 min in the dark, and then analyzed by flow cytometry (Thermo Fisher, USA) and FlowJo software.

### Cell Uptake Assay of MOF

1 mg Ce‐MOF was mixed thoroughly with 1 mL of PBS under sonication and 4 mL of fluorescein‐5‐maleimide (MedChemExpress, USA) standard solution (5 mg mL^−1^) was added. The stirrer was added and left at room temperature overnight at 1400 rpm conditions. Subsequently the MOF was washed five times with PBS for 3 min each to remove unbound dye. The fluorescently labeled MOF was synthesized into a hydrogel as described above and cocultured with raw cells for 1 and 3 d, and with neural cells for 3 and 7 d. The cells were fixed for 30 min, treated with 0.3% Triton X‐100 for 15 min, and then stained with rhodamine (Yeasen, China) and DAPI (Yeasen, China). Cells were imaged with a fluorescence microscope

### Mitochondrial Function

MMP values of macrophages were determined using the JC‐1 MMP assay kit (MedChemExpress, USA). Cells were incubated using jc‐1 staining working solution for 20 min at 37 °C and subsequently washed twice with PBS. Images were captured with a fluorescence microscope (Carl Zeiss, USA). MMP (ratio of red/green fluorescence intensity) was calculated using ImageJ software.

The intracellular and mitochondrial ROS were determined using DCFH‐DA (MedChemExpress, USA) and MitoSox (MedChemExpress, USA) probe. Cells were incubated with working solution with 5 × 10^−6^ m DCFH‐DA or 5 × 10^−3^ m MitoSOX for 30 min at 37 °C. They were subsequently examined by fluorescence microscopy and flow cytometry respectively as mentioned above.

### Seahorse Assay

The Seahorse Extracellular Flux XFe24 analyzer (Agilent, USA) was used to investigate the mitochondrial oxygen consumption rates (OCRs) and extracellular acidification rates (ECARs). Cells were seeded at 1 × 10^5^ cells per well in Seahorse XF‐24 plates cultured in each hydrogel leachate. The cell plate was first incubated in the CO_2_‐free incubator for 1 h before analysis. For ECAR determination, glucose (10 × 10^−3^ m), Oligo (1 × 10^−6^ m) and 2‐DG (50 × 10^−3^ m) were sequentially injected into cells. The glycolysis was evaluated as the maximal ECAR value before Oligo treatment minus last ECAR value before glucose injection; the glycolytic capacity was evaluated as the maximal ECAR value after Oligo injection minus last ECAR value before glucose injection; and glycolytic capacity minus glycolysis represent glycolytic reserve. For OCR analysis, Oligo (1 × 10^−6^ m), FCCP (1.5 × 10^−6^ m), and rotenone/antimycin (R/A) (2 × 10^−6^ m) were injected sequentially into cells, and the basal respiration was evaluated as the last OCR before Oligo injection minus non‐mitochondrial respiration; the maximal respiration was evaluated as the maximum OCR after FCCP injection minus minimum OCR after R/A injection; and ATP production was evaluated as the final OCR prior to Oligo injection minus minimum OCR after Oligo injection.

### Transcriptomics and Metabolomics

Macrophages cocultured with different nanoparticles were collected. For transcriptomics, the total RNA was extracted using an RNA purification kit (Thermo Fisher, USA), and for metabolomics, cells were put into liquid nitrogen for rapid freezing for 5 min for metabolite extraction. The transcriptomics sequencing and metabolomics LC‐MS analysis were performed by Panomix Biomedical Tech Co. For each pairwise comparison, genes or metabolite with false discovery rate (FDR) <0.05 were considered significant and log2‐fold changes of expression between conditions (logFC) were reported. Gene Ontology (GO) and Kyoto Encyclopedia of Genes and Genomes (KEGG) enrichment analysis were used to determine the biological functions or pathways that differential transcripts primarily affect.

### ELISA

Macrophages were cultured with different hydrogels after 12 h stimulation with LPS (the control group was not stimulated) for five days, and then, the supernatant of the cell culture medium was collected. The levels of inflammatory cytokines (TNF‐a, IL‐6) and those of growth factors TGF‐b, PDGF, and NT3 in cell culture supernatant were detected by ELISA kits (SAB, USA).

### Rats Spinal Cord Hemisection Model

SD male rats (200–250 g) were obtained from Zhaoyan (Suzhou) New Drug Research Center Co., Ltd. The Ethics Committee of Soochow University approved all surgical operations and perioperative treatments (SUDA20230905A02). SD rats (6–8 weeks old) were anesthetized by intraperitoneal injection of 2% sodium pentobarbital (50 mg kg^−1^). A longitudinal incision was made in the back of the rat centered on T9, and after careful separation of the paravertebral muscles, the T9 vertebral plate was completely exposed in the spinal canal. The right vertebral plate was opened to expose the spinal cord, and the right side of the spinal cord was cut with a medical blade to form a 3 mm hemispheric spinal cord defect. Penicillin (2 × 10^5^ units, KEDA, China) was injected intramuscularly daily for 7 d, and the bladder was manually emptied every 12 h. The sham‐operated group underwent simple laminectomy, and the blank control group underwent spinal hemilaminectomy without implanted material. The negative control group was implanted with different hydrogels.

### Analysis of Locomotor Function and Urinary Bladder Function

The open field Basso Mouse Scale (BMS) locomotor test and footprint analysis were used to evaluate the locomotion recovery. Rats were allowed to walk on an inclined plate and the BMS scores were judged based on hind limb locomotor function ranging from 0 (no ankle movement) to 9 (complete function recovery). For the footprint analysis, the fore and hind limbs of rats were dipped into blue and red ink respectively, and then the rats were allowed to walk on narrow paper. The frequency of toe drag is characterized as the ratio of drag to total footprint. To assess bladder function, maximum bladder wall thickness was measured by staining bladder tissue sections with HE staining.

### Motor Evoked Potentials (MEPs) Detection

The MEPs data were recorded with the Powerlab 4/26 (ADInstruments, Australia) from rats 6 weeks after SCI. Rats were anesthetized by intraperitoneal injection of 2% sodium pentobarbital (50 mg kg^−1^) and then fixed on a board. The stimulating electrode was inserted into the spinal cord above the site of injury, the grounding electrode was inserted into the tail of the mouse, and motor evoked potentials were recorded with a recording electrode inserted into the ipsilateral gastrocnemius muscle. The stimulation strength was 0.5 V and the stimulus duration was 500 ms.

### In Vivo ROS Levels Detection

The in vivo ROS levels in rats 7 d after SCI were probed with ROS Brite 700 (AAT Bioquest, USA). Rats were first anesthetized with isoflurane and ROS Brite 700 (in vivo imaging solutions, 100 × 10^−6^ m in Hanks with 20 × 10^−3^ m HHBS) were injected with into the lesion site. Bioluminescence images were acquired with an in vivo imaging system (Spectral Instruments Imaging, USA).

### Histological Analysis

Euthanasia of rats by 2% sodium pentobarbital injection followed by intracardiac perfusion fixation with PBS and 4% paraformaldehyde. The spinal cord was embedded in paraffin and then cut into 20 µm thick sections using electric slicer (Leica, Germany). HE and immunofluorescence staining using primary antibody including anti‐iNOS antibody (GB11119, Servicebio, China), anti‐F4/80 antibody (GB113373, Servicebio, China), anti‐ARG1 antibody (GB11285, Servicebio, China), anti‐CD31 antibody (GB11063, Servicebio, China), anti‐Tuj‐1 antibody (GB12139, Servicebio, China), anti‐GFAP antibody (GB11096, Servicebio, China), anti‐GAP43 antibody (GB11095, Servicebio, China), anti‐NG2 antibody (GB115534, Servicebio, China), anti‐TOMM20 antibody (GB111481, Servicebio, China), anti‐p‐P70S6K antibody (AP0564, Abclonal, China), anti‐NeuN antibody (Servicebio, China) in samples were used to evaluate lesion cavity and nerve regeneration.

### Statistical Analysis

Data are shown as mean ± standard deviation (SD). GraphPad Prism 8.2 and Origin 9.1 were used to acquire graphics and perform statistical analysis. Differences between groups were tested by one‐way or two‐way ANOVA and Tukey's multiple comparison method. *P* < 0.05 was considered to indicate statistical significance.

## Conflict of Interest

The authors declare no conflict of interest.

## Author Contributions

X.J., W.W., J.T., and M.H. contributed equally to this work. K.X., Y.G., and L.C. designed the experiments. X.J., W.W., and M.H. synthesized the materials. Y.X. and L.Z. assisted in characterizing the materials. X.J., J.T., and J.W. performed in vitro and in vivo experiments and analyzed the data. Y.H., Z.D., and H.S. provided good advice for the cell and animal experiments. X.J., W.W., and J.T. wrote the paper.

## Supporting information

Supporting Information

## Data Availability

The data that support the findings of this study are available from the corresponding author upon reasonable request.
