# Supplementary material for: Ligand‐Screened Cerium‐Based MOF Microcapsules Promote Nerve Regeneration via Mitochondrial Energy Supply
Source: Adv Sci (Weinh). 2023 Nov 30;11(6):2306780. doi: 10.1002/advs.202306780 (PMC10853750; doi:10.1002/advs.202306780)
Supplement: Supplementary file 1 — Supporting Information [file ADVS-11-2306780-s001.pdf]

ADVANCED SCIENCE
Open Access

Supporting Information

for *Adv. Sci.*, DOI 10.1002/advs.202306780

Ligand-Screened Cerium-Based MOF Microcapsules Promote Nerve Regeneration via Mitochondrial Energy Supply

Xinzhao Jiang, Wei Wang, Jincheng Tang, Meng Han, Yichang Xu, Lichen Zhang, Jie Wu, Yiyang Huang, Zhouye Ding, Huiwen Sun, Kun Xi, Yong Gu* and Liang Chen**

Supplementary Materials for

Ligand-screened Cerium-based MOF Microcapsules promote Nerve Regeneration via Mitochondrial Energy Supply

Authors

Xinzhao Jiang[1]†, Wei Wang[1]†, Jincheng Tang[1]†, Meng Han[1,2]†, Yichang Xu[1], Lichen Zhang[1], Jie Wu[1], Yiyang Huang[1], Zhouye Ding[1], Huiwen Sun[1], Kun Xi[1]*, Yong Gu[1]*, Liang Chen[1]*

Affiliations

[1]Department of Orthopedics, The First Affiliated Hospital of Soochow University, Orthopedic Institute, Soochow University, 188 Shizi Road, Suzhou, Jiangsu, China.
[2]Department of Spinal Surgery, Xuzhou Central Hospital, Xuzhou, China.

† These authors contributed equally to this work.

* Corresponding author: Kun Xi, sudaxk@163.com; Yong Gu, guyongsuzhou@163.com; Liang Chen, chenliang1972@sina.com

This file includes:

Figures. S1 to S23
Table S1

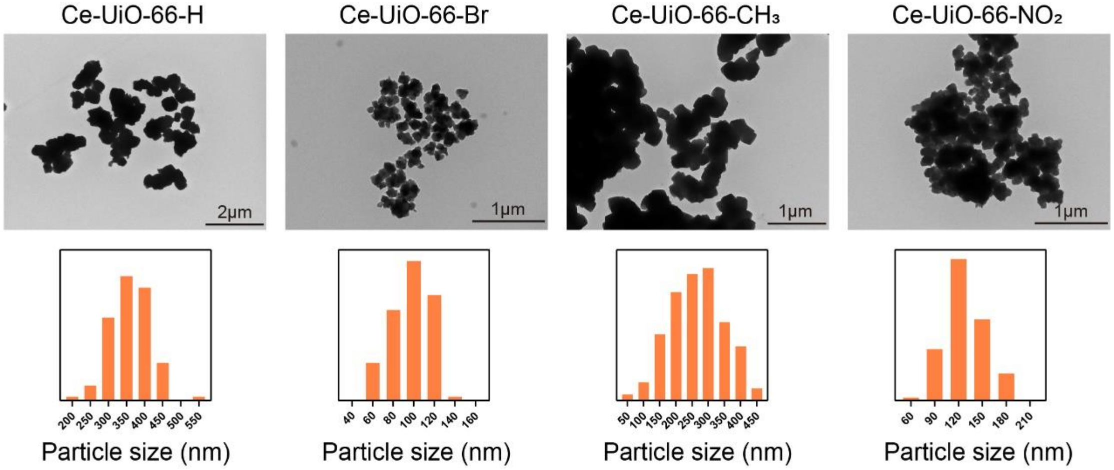

Figure. S1. TEM images and particle sizes of Ce-UiO-66-X

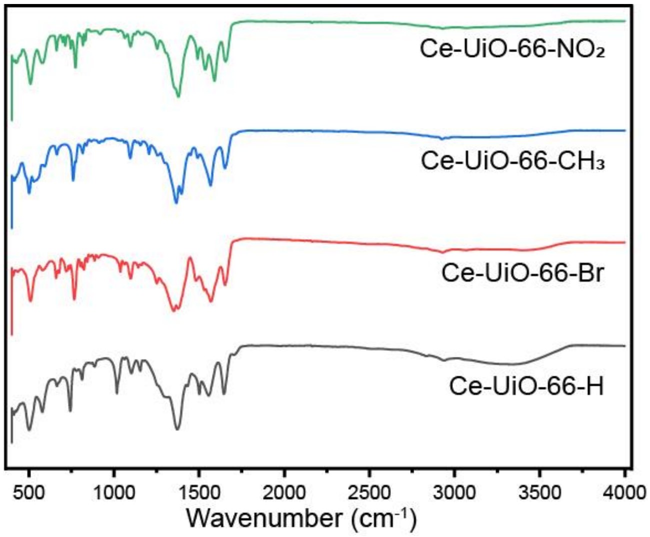

Figure. S2. FTIR (Fourier Transform infrared spectroscopy) of Ce-UiO-66-X

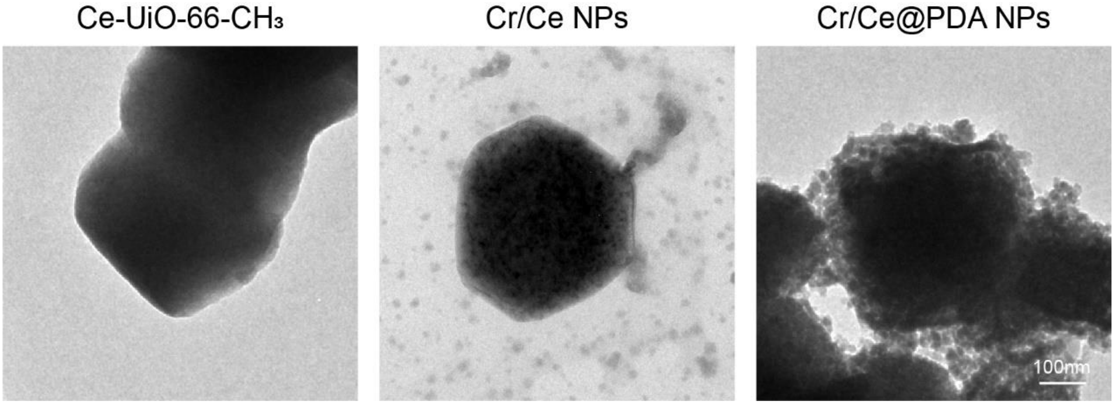

Figure. S3. TEM images of Ce-MOF, Ce/Cr NPs and Ce/Cr@PDA NPs.

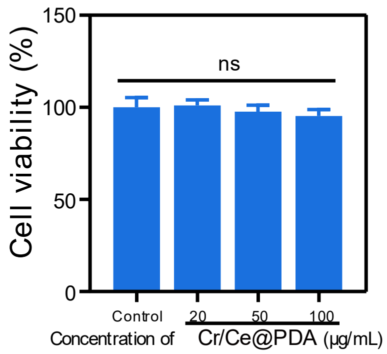

Figure. S4. Cell viability under different concentrations of Ce/Cr@PDA NPs. (n = 3, error bars, means ± SD; all analyses were done using one-way ANOVA with Tukey's post hoc test, ns no significance)

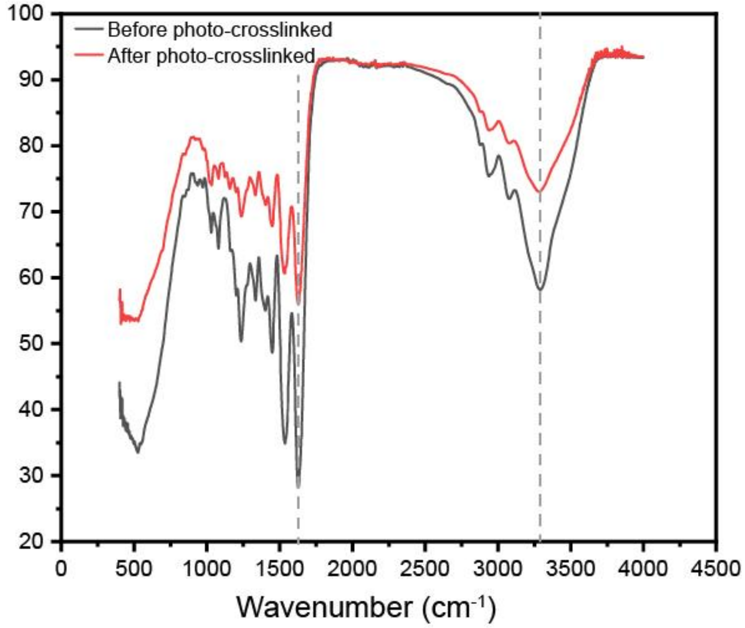

Figure. S5. FTIR of hydrogel before and after photo-crosslinked.

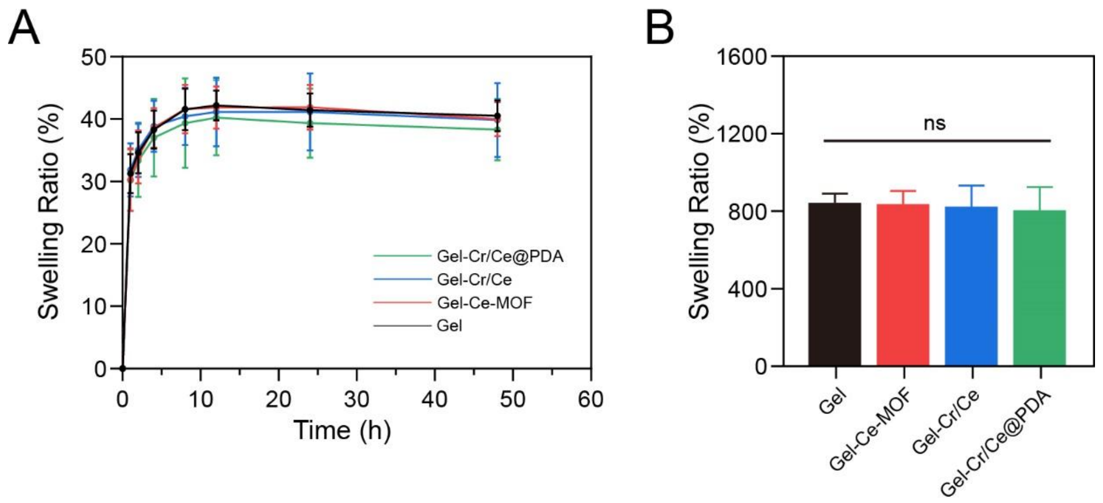

Figure. S6. Swelling performance of hydrogels. (n = 3, error bars, means ± SD; all analyses were done using one-way ANOVA with Tukey's post hoc test, ns no significance)

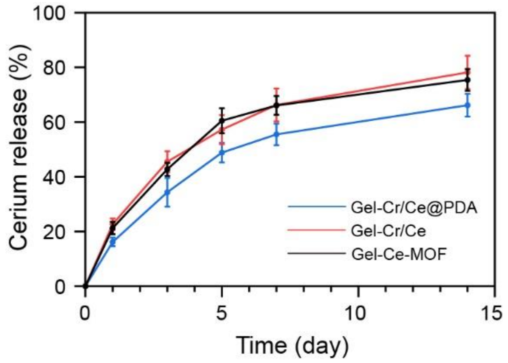

Figure. S7. Cerium release curve of the hydrogels.

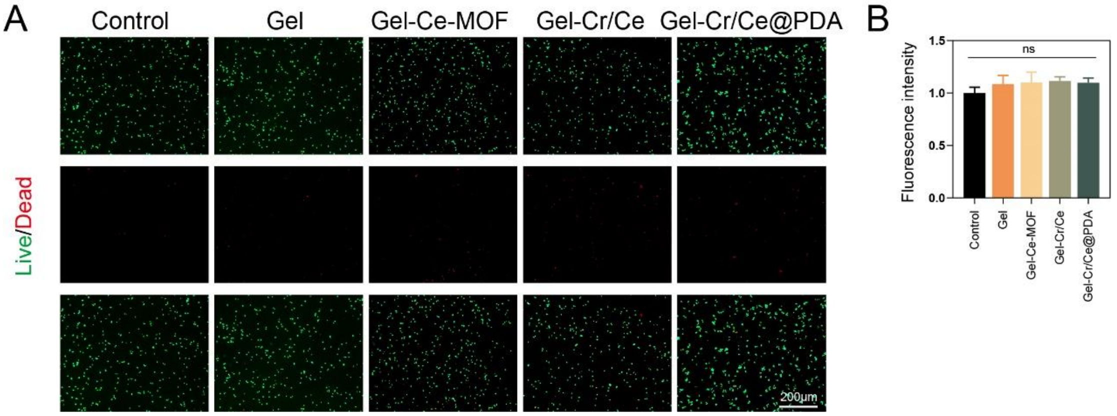

Figure. S8. Live/dead staining images (A) and semiquantitative analysis (B) of cells co-cultured with hydrogels after 5 days. (n = 3, error bars, means ± SD; analysis was done using one-way ANOVA with Tukey's post hoc test, ns no significance)

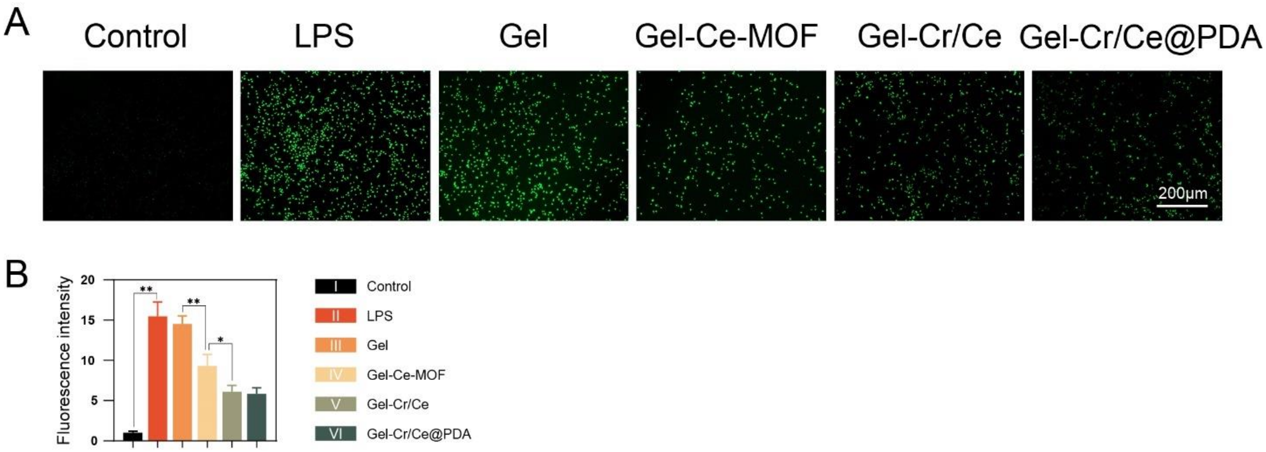

Figure. S9. Fluorescent images (A) and semiquantitative analysis (B) of DCFHDA-labeled macrophages (n = 3, error bars, means ± SD; analysis was done using one-way ANOVA with Tukey's post hoc test *P < 0.05 and **P < 0.01.)

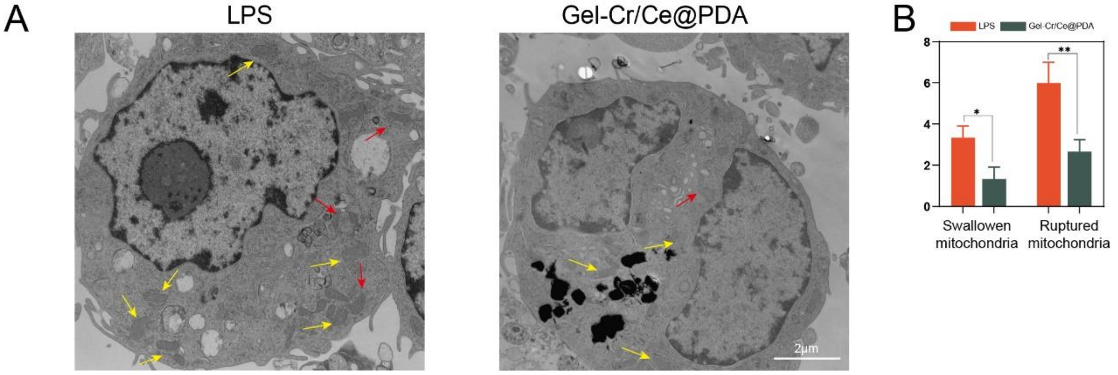

Figure. S10. Bio-TEM images (A) and quantitative analysis (B) of swollen or ruptured mitochondria (Swollen mitochondria marked with yellow arrows, and ruptured mitochondria marked with red arrows). (n = 3, error bars, means ± SD; the analysis was done using unpaired two-tailed t test *P < 0.05 and **P < 0.01.)

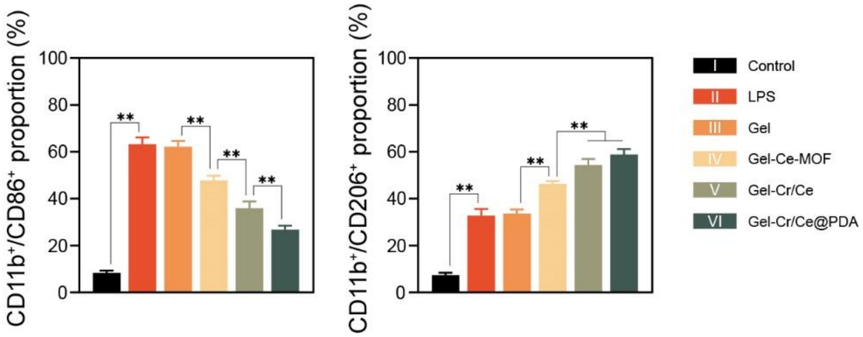

Figure. S11. The proportion of $CD80^+$ and $CD206^+$ cells in the flow analysis. (n = 3, error bars, means ± SD; analysis was done using one-way ANOVA with Tukey's post hoc test *P < 0.05 and **P < 0.01.)

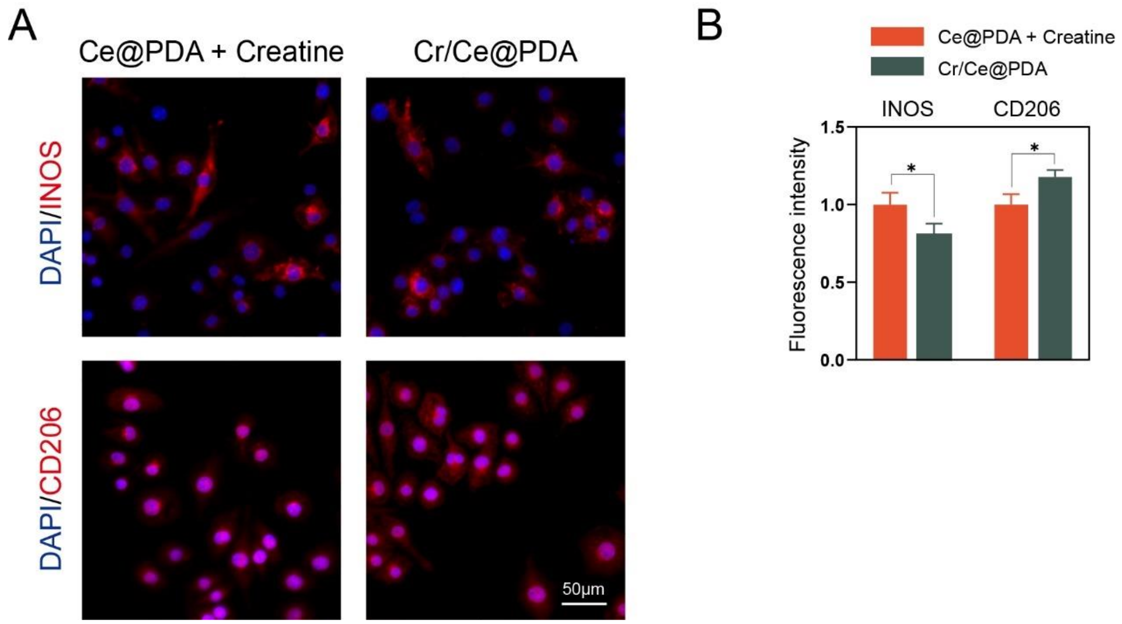

Figure. S12. The immunofluorescence images (A) and quantitative analysis (B) of INOS and CD206 expression in Ce@PDA&creatine and Cr/Ce@PDA treated macrophages. (n = 3, error bars, means ± SD; the analysis was done using unpaired two-tailed t test *P < 0.05 and **P < 0.01.)

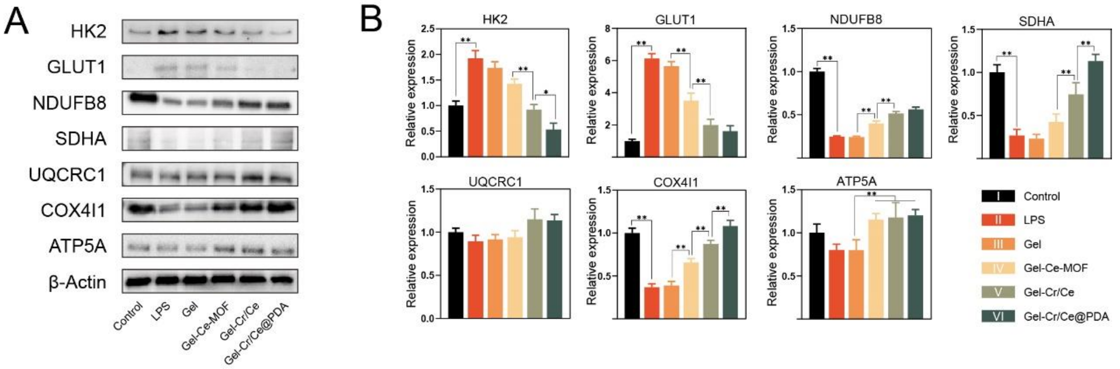

Figure. S13. Western blot (A) and semiquantitative analysis (B) of the glycolysis and OXPHOS marker proteins. (n = 3, error bars, means ± SD; analysis was done using one-way ANOVA with Tukey's post hoc test $*P < 0.05$ and $**P < 0.01$.)

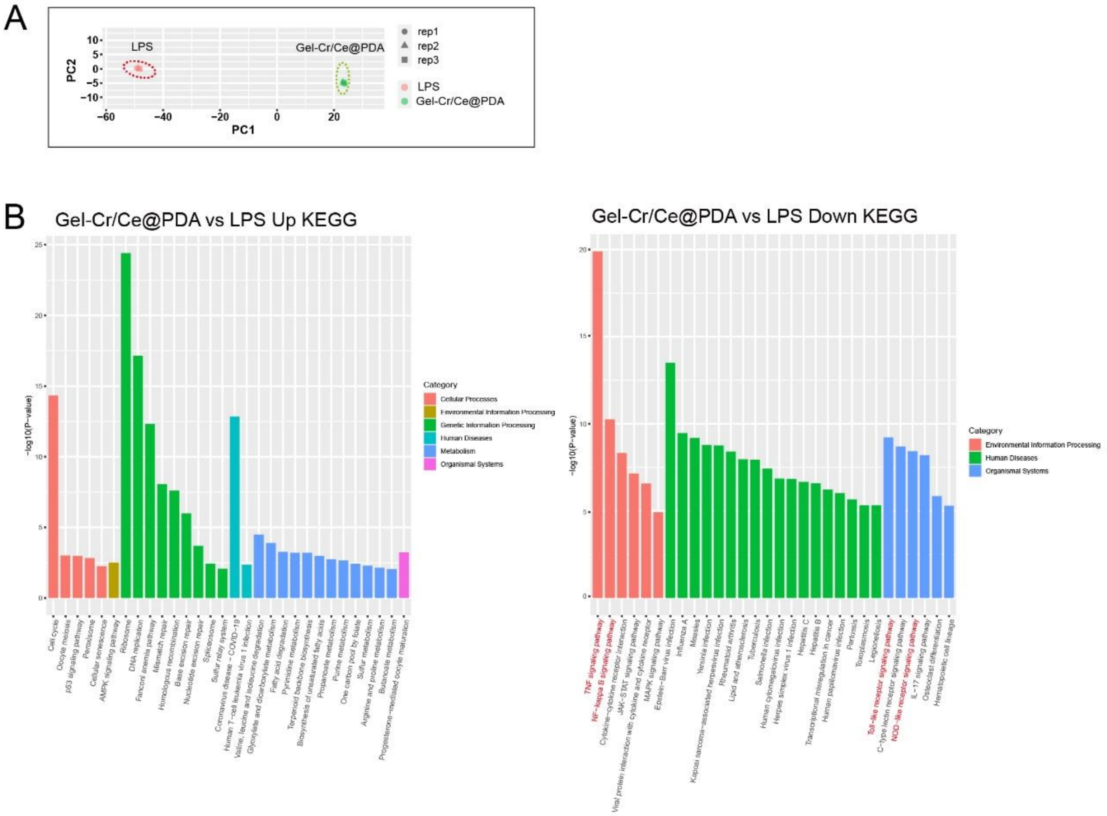

Figure. S14. (A) Principal component analysis (PCA) of transcriptomic analysis. (B) TOP30 up and down KEGG pathways of transcriptomic analysis.

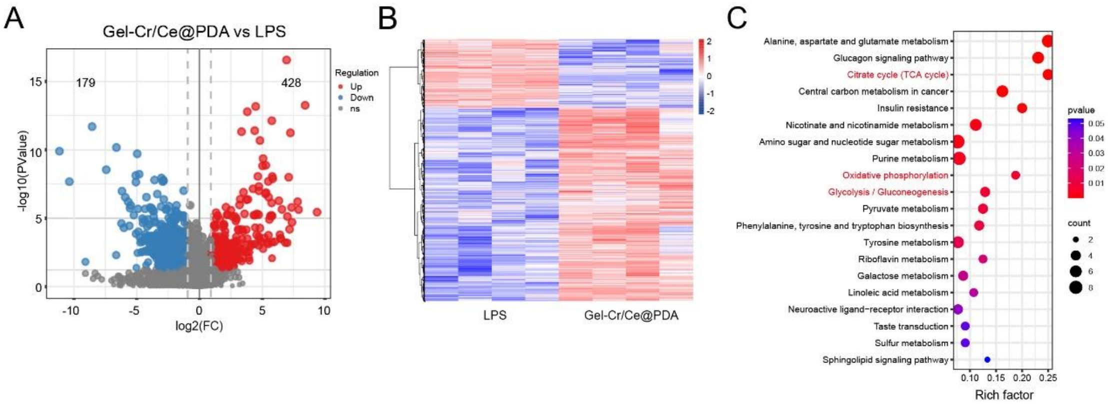

Figure. S15. (A)Volcano map, (B)heatmap, and (C)TOP 20 KEGG pathways analysis of metabolites.

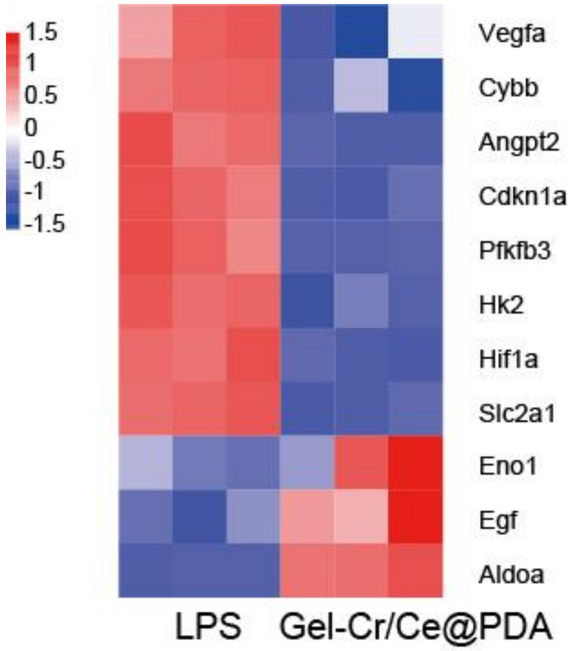

Figure. S16. Heatmap of the genes related with HIF signaling pathway

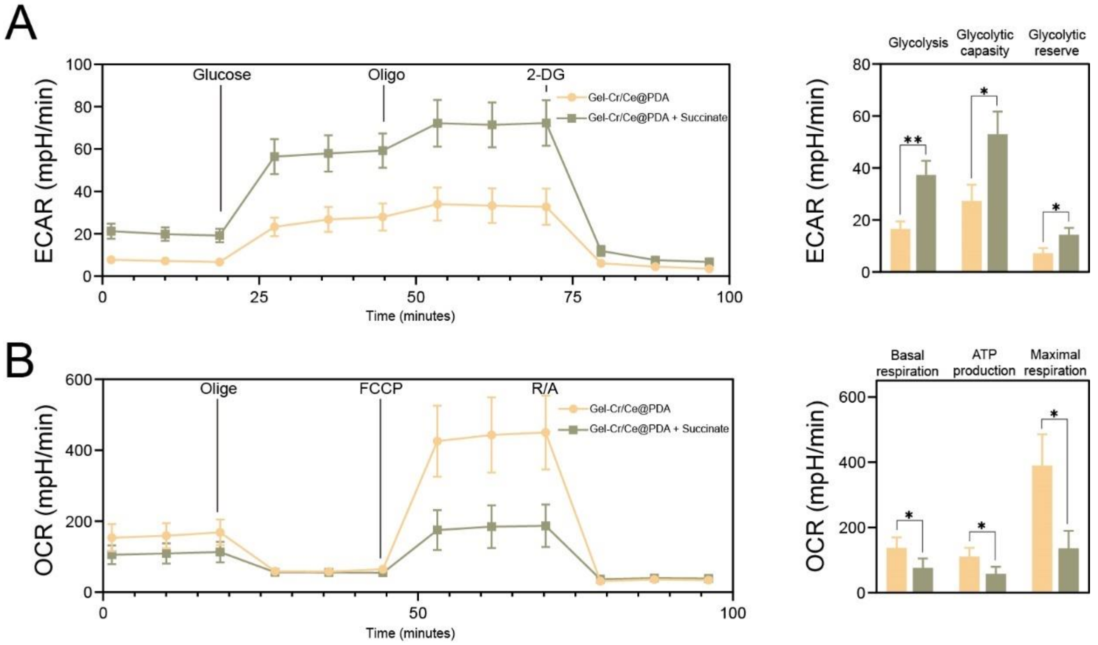

Figure. S17. (A) Real-time ECARs of macrophages in the glycolysis stress test and semiquantitative analysis of glycolysis, glycolytic capacity and glycolytic reserve. (B) Real-time OARs of macrophages in the cell mitochondrial stress test and semiquantitative analysis of basal respiration, maximal respiration and ATP production. (n = 3, error bars, means ± SD; the analysis was done using unpaired two-tailed t test *$P < 0.05$ and **$P < 0.01$.)

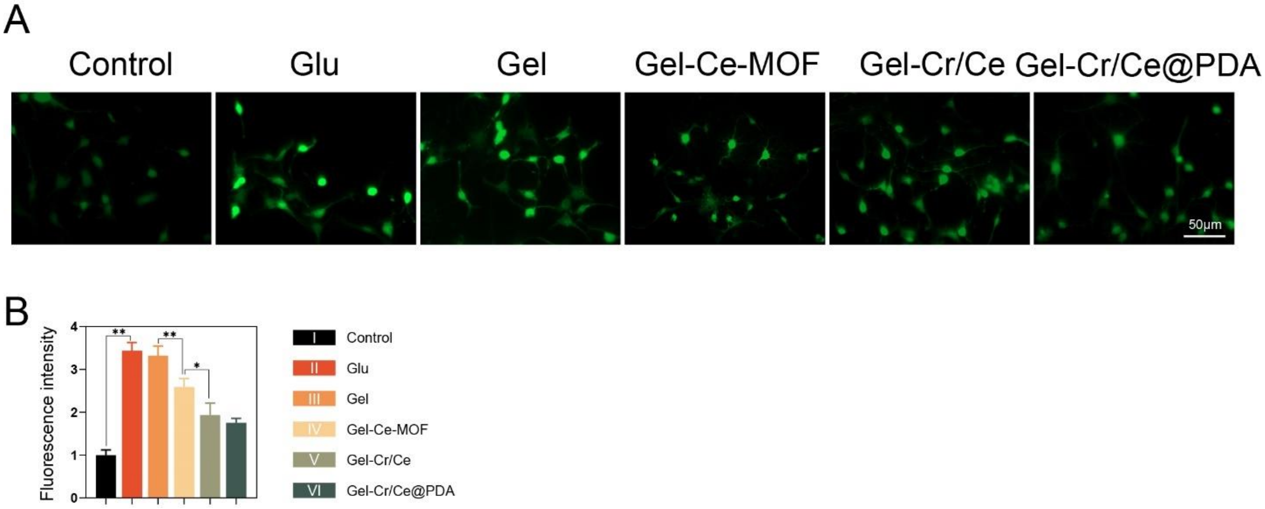

Figure. S18. Fluorescent images and semiquantitative analysis of DCFHDA-labeled neuronal cells. (n = 3, error bars, means ± SD; analysis was done using one-way ANOVA with Tukey's post hoc test *P < 0.05 and **P < 0.01.)

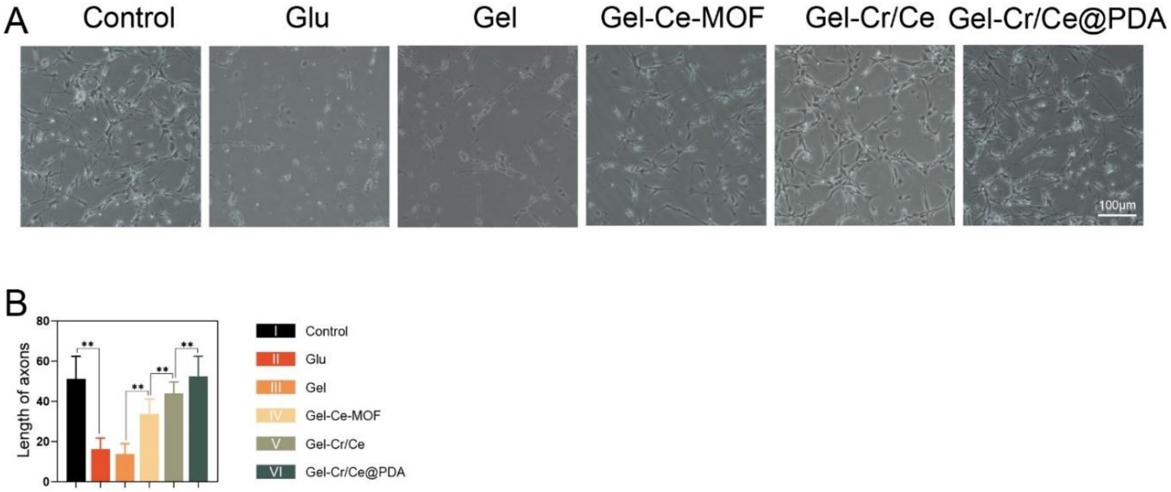

Figure. S19. Microscopy images and quantitative analysis of axon outgrowth in different groups. (n = 3, error bars, means ± SD; analysis was done using one-way ANOVA with Tukey's post hoc test *P < 0.05 and **P < 0.01.)

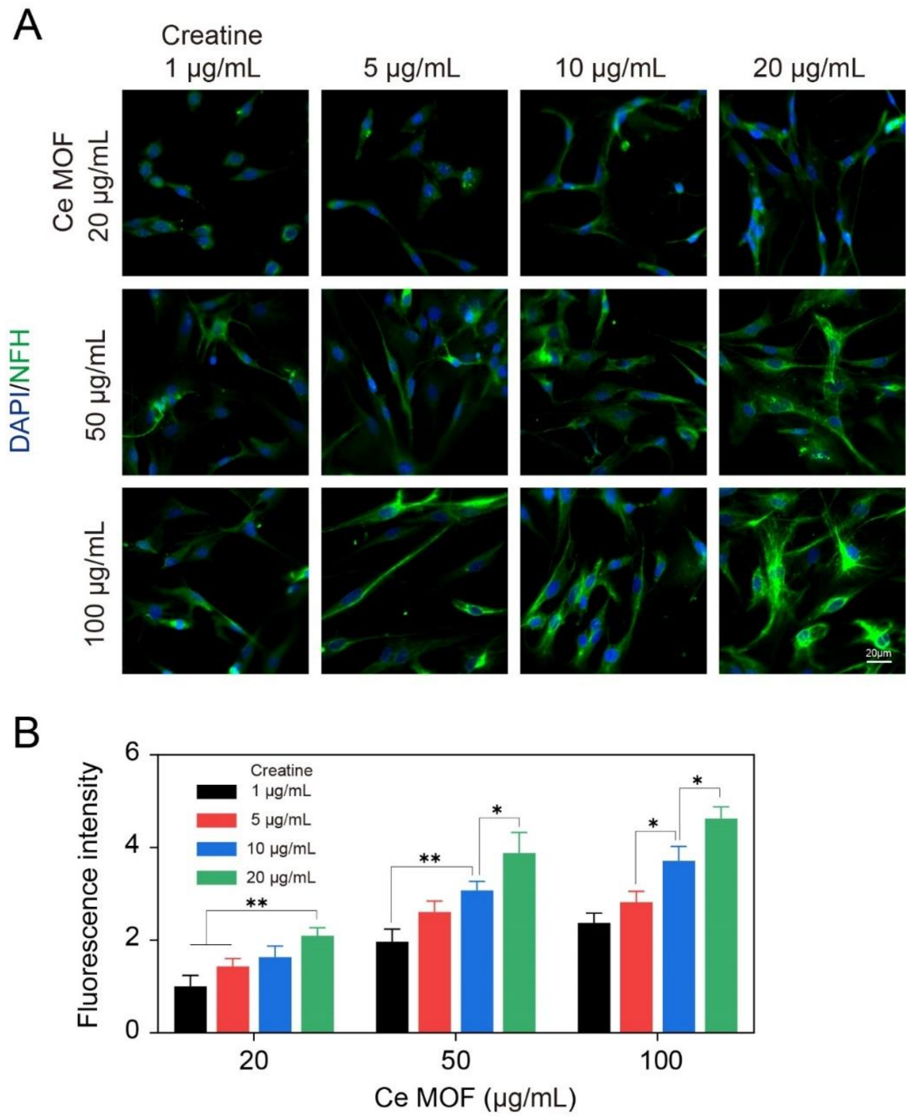

Figure. S20. Fluorescence images (A) and quantitative analysis (B) of neural cells cultured with with different concentrations of cerium MOF and creatine (n = 3, error bars, means ± SD; all analyses were done using one-way ANOVA with Tukey's post hoc test, *P < 0.05 and **P < 0.01.).

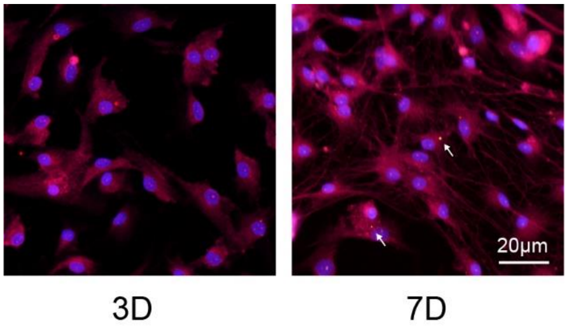

Figure. S21. Fluorescence images of neural cells cultured with fluorescein labelled Ce/Cr@PDA NPs for 3 and 7 days.

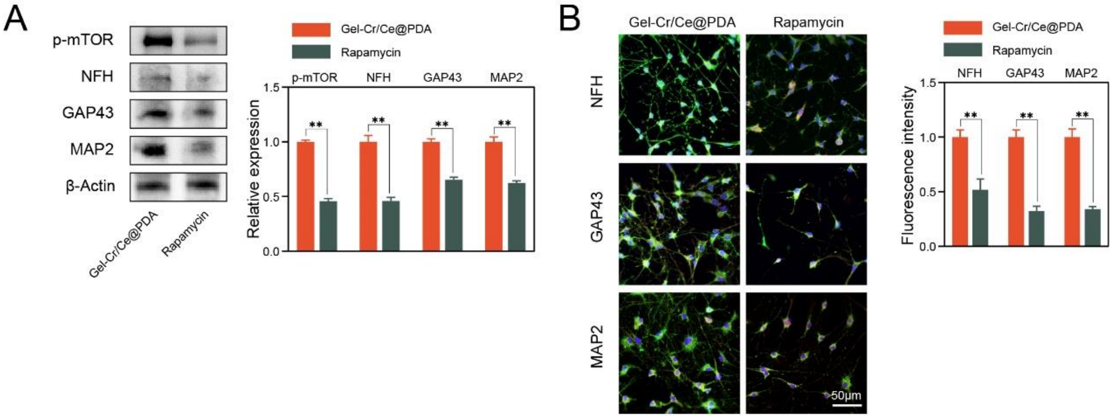

Figure. S22. Western blot and immunofluorescence of neurogenic markers expression in neuronal cells after p-mTOR inhibition. (n = 3, error bars, means ± SD; the analysis was done using unpaired two-tailed t test $*P < 0.05$ and $**P < 0.01$.)

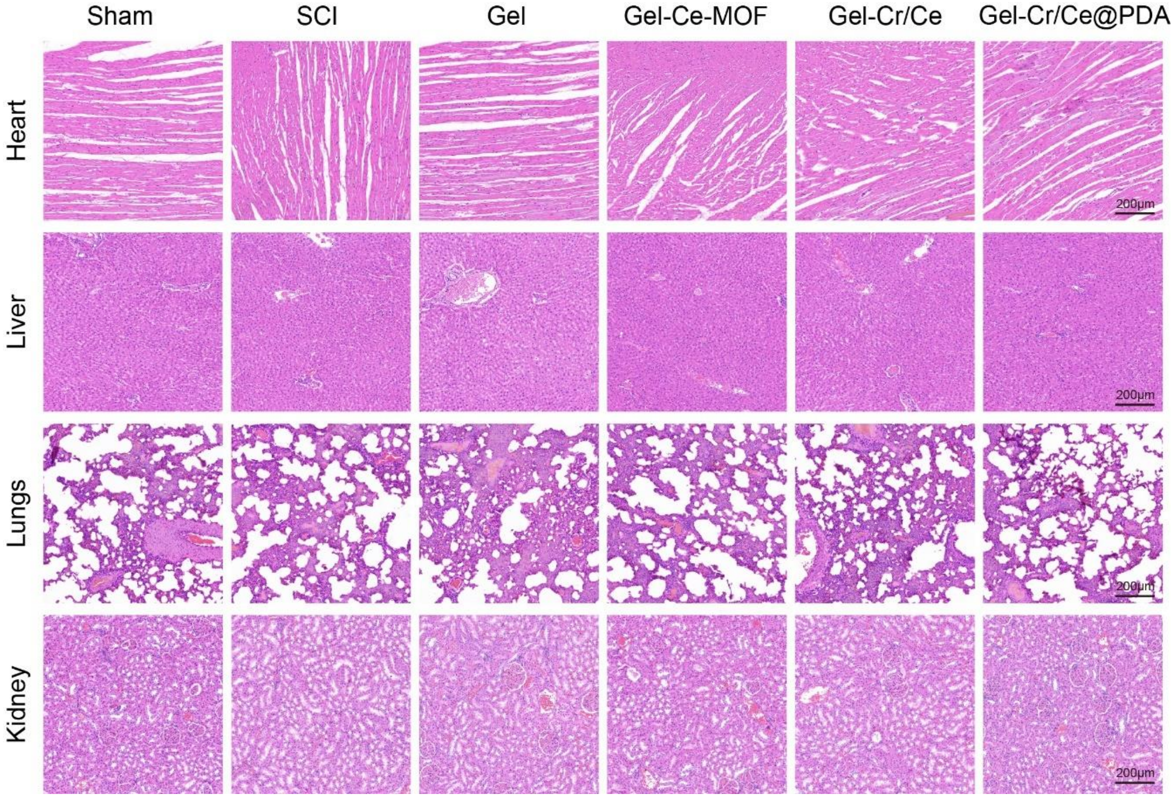

Figure. S23. Representative images of H&E staining of main organs of SCI rats in different groups.

	Ce-UiO-66-H	Ce-UiO-66-Br	Ce-UiO-66-CH_3	Ce-UiO-66-NO_2
Surface area (m^2/g)	1055.52	164.29	770.12	346.89
Pore volume (ml/g)	0.39	0.06	0.38	0.14
Loading capacity	14.40%±0.55%	6.38%±0.38%	13.92%±0.32%	9.24%±0.35%

Table. S1. Surface area, pore volume and creatine loading capacity of Ce-UiO-66-X
